# Disentangling the Influence of Socioeconomic Risks on Children's Early Self‐Control

**DOI:** 10.1111/jopy.12288

**Published:** 2016-12-10

**Authors:** Terry Ng‐Knight, Ingrid Schoon

**Affiliations:** ^1^ University College London Institute of Education; ^2^ Social Science Center Berlin (WZB)

**Keywords:** Self‐control, self‐regulation, effortful control, socioeconomic risk, temperament

## Abstract

**Objective:**

Previous studies have shown that individual differences in self‐control emerge early in childhood and predict a range of important outcomes throughout childhood and adulthood. There is, however, less knowledge about the social origins of self‐control, including the mechanisms by which early socioeconomic adversity may lead to lower levels of self‐control. This study aimed to extend understanding of the link between socioeconomic adversity and self‐control by (a) testing which individual aspects of socioeconomic risk uniquely predict lower self‐control; (b) testing whether objective socioeconomic risk operates independently of, or via, subjective parental stress; and (c) examining the interplay of socioeconomic risk factors and individual differences in children's temperament as predictors of early self‐control.

**Method:**

Data were from a UK population birth cohort of 18,552 children born in 2000 and 2001.

**Results:**

Multiple individual socioeconomic risk factors have independent associations with children's self‐control, including low parental education, income, and occupational class; insecure housing tenure; and younger parenthood. Results point to independent additive effects of exposure to objective and subjective risk. There was evidence of mothers' subjective stress partially mediating objective socioeconomic risks but only weak evidence of hypothesized interaction effects between temperament and socioeconomic risk.

**Conclusions:**

Results were consistent with additive risk and bioecological perspectives.

The ability to self‐regulate attention, emotions, and behavior is central to positive functioning throughout the life course and is associated with a range of developmental outcomes in later life (Moffitt et al., 2011). The acquisition of these abilities during childhood can be considered core indicators of developmental health (Kopp, 1982). Given the key role of self‐control—a core component of the broader conscientiousness trait—in development and successful life outcomes, improving our understanding of its early developmental precursors is an important research aim (Caspi, Roberts, & Shiner, 2005; Eisenberg, Duckworth, Spinrad, & Valiente, 2014). In this study, we focus on the role of socioeconomic risks, disaggregating their unique influence and examining the interplay between socioeconomic resources and individual differences in early temperament in shaping variations in levels of self‐control.

## An Integrative Model of Self‐Control

Following recent calls for more integrative models of self‐regulation, we draw on research on the related constructs of self‐control and effortful control, which both refer to internal processes grounded in components of executive functioning that facilitate individuals' abilities to plan, focus and shift attention, inhibit inappropriate responses, and activate behavior to pursue goals (Eisenberg et al., 2014; Zhou, Chen, & Main, 2012). Distinctions between self‐control and effortful control have generally relied on theoretically proposed differences, where effortful control is defined as an innate predisposition toward good self‐regulation and self‐control is viewed as a broader personality trait that emerges, in part, from effortful control (Diamond, 2013). However, both self‐control and effortful control are believed to be influenced by social as well as biological factors (including genes), and there is, as yet, no reliable way of distinguishing potentially innate self‐control behaviors from those which are learned or socialized. Given that both constructs draw heavily on inhibitory control, a core executive function involving control of “one's attention, behaviour, thoughts and/or emotions to override a strong internal predisposition or external lure, and instead do what's more appropriate” (Diamond, 2013, p.137), it seems suitable to study the antecedents of these early self‐control behaviors under the broader framework of self‐regulatory abilities, as has been proposed elsewhere (Zhou et al., 2012).

Self‐control behaviors and traits emerge throughout the first few years of life and show first signs that they can be reliably measured at about age 3 years (Kochanska & Knaack, 2003; Kochanska, Murray, & Harlan, 2000; Kopp, 1982). The ability to control attention and behavior improves with age for most children, with fewer difficulties reported as children age and large improvements shown between ages 2 and 3 years (Carlson, Mandell, & Williams, 2004; Kochanska et al., 2000). However, the pace of improvements tends to slow after these early years, and existing research indicates self‐control shows moderate rank‐order stability during both early and middle childhood (Carlson et al., 2004; Jones, Rothbart, & Posner, 2003; Kochanska & Knaack, 2003). Therefore, the early years appear to be an important period for the acquisition of self‐control, but there remains more to be learned about the underlying processes.

## Exposure to Socioeconomic Adversity

A key risk factor undermining the development of self‐control is socioeconomic adversity, with exposure to social‐contextual risk thought to be particularly detrimental to children's development during the first few years of life (Choe, Olson, & Sameroff, 2013). Empirical associations between socioeconomic disadvantage and lower childhood self‐control have been found in multiple studies using different measures of disadvantage, including low parental income, low parental education, and cumulative risk indexes (e.g., Lengua, 2009; Lengua, Honorado, & Bush, 2007; Lengua et al., 2015; Li‐Grining, 2007; Mistry, Benner, Biesanz, Clark, & Howes, 2010). These observations are consistent with allostasis theory, which suggests constant exposure to ecological risk strains the flexibility of children's self‐regulatory competence by placing continuous demands on the neuroendocrine systems that influence how children respond to stress and regulate behavior, attention, and emotions (Evans & Schamberg, 2009).

While there are strong empirical and theoretical links between socioeconomic disadvantage and lower self‐control, a number of gaps remain in the knowledge base. First, it has been noted that it is not yet clear which specific aspects of socioeconomic risk influence children's self‐control (Lengua et al., 2007, 2015; Raver, Blair, & Willoughby, 2013). Socioeconomic risk factors include those related to parental socioeconomic status (SES) such as low education, low income, low occupational class, and unemployment; family structure characteristics such as single parenthood, family size, and young maternal age; and residential risks such as overcrowding and insecure housing tenure (Evans, 2006; Kiernan & Huerta, 2008; Li‐Grining, 2007). Many of these risk factors are correlated and therefore require large samples to adequately examine them simultaneously. Previous research has generally relied on cumulative risk indexes that account for the co‐occurrence of risk factors, but also have the limitation of weighting all factors equally and obscuring inferences about the relative role of individual risk factors (Lengua et al., 2007). In the current study, we utilize data from a large, representative birth cohort to overcome these limitations and to advance understanding of the unique associations between socioeconomic risk factors and early self‐control. Moreover, we examine potential mediating processes linking early exposure to socioeconomic risk factors and children's early self‐control, focusing on the burden of parental stress associated with the accumulation of risks.

## Processes Linking Socioeconomic Risk and Early Self‐Control

There is evidence to suggest that socioeconomic adversity, such as the experience of poverty, is associated with experiences of financial strain, often operationalized through parents' subjective experience of not being able to manage financially (McLoyd, 1998), as well as psychological distress (Newland, Crnic, Cox, & Mills‐Koonce, 2013). The family stress model is a leading perspective proposed to explain how socioeconomic adversity and associated strain influence children's development (Conger, Conger, & Martin, 2010).

Like other aspects of children's cognitive and behavioral development, the negative effects of socioeconomic disadvantage on children's self‐control are thought to partially act through more proximal family stress mechanisms, including parents' experience of financial and emotional strain, which in turn shape parent–child interactions (Eisenberg et al., 2005; Kiff, Lengua, & Zalewski, 2011; Mistry et al., 2010). Given the allostatic stress mechanisms that are supposed to underlie socioeconomic disadvantage (Blair, 2010; Evans & Schamberg, 2009), it is important to test whether indicators of subjective psychological strain often experienced by socioeconomically disadvantaged families can be empirically differentiated from objective measures of socioeconomic disadvantage in predicting children's self‐control. In particular, the question of whether indicators of socioeconomic disadvantage are uniquely associated with children's self‐control after controlling for parental perceptions of financial strain needs to be addressed (Raver et al., 2013).

The family stress model has been widely tested and largely confirmed across different cultural contexts (Conger et al., 2010). Moreover, theoretical developments have moved beyond earlier assumptions about the singular direction of effects to new interactionist perspectives regarding the interplay among individual differences, socioeconomic resources, and family processes. In this article, we examine these relationships more closely, especially the interactive relationships between socioeconomic resources and individual differences in early temperament that may shape variations in levels of self‐control. In particular, we ask whether children's temperamental predispositions play a role in magnifying or attenuating the effects of exposure to socioeconomic adversity on subsequent levels of self‐control.

## Interplay Between Socioeconomic Risk and Infant Temperament

Individual differences in self‐control are associated with biological factors as well as the socioeconomic factors described above. In particular, psychobiological theories suggest that self‐control has a biological basis in infant temperament (Rothbart, Derryberry, & Posner, 1994), and this is supported by empirical findings showing associations between early temperamental “difficulties” and lower levels of later self‐regulation (Feldman, Greenbaum, & Yirmiya, 1999; Kochanska & Knaack, 2003; Kochanska et al., 2000). Broadly speaking, developmental theories suggest children's development is driven by both social‐contextual factors (the social causation perspective) and characteristics of the individual (the social selection perspective), as well as the dynamic interplay of these factors over time (the interactionist perspective; Bronfenbrenner & Morris, 2006; Conger et al., 2010; Sameroff, 2009; Schoon, Sacker, & Bartley, 2003). However, although temperament and socioeconomic risk are both considered important antecedents of children's self‐control, less is known about how these factors operate alongside each other.

First, most existing studies have focused on the predictive role of either socioeconomic risk or of infant temperament, leaving it unclear whether these two sets of risk factors have unique associations with children's self‐control. One study that did include measures of both factors found evidence of associations for socioeconomic risks and not for infant temperament, though the authors note that the null findings may be due to the limited measure of infant temperament available in their study (Li‐Grining, 2007). Given that aspects of socioeconomic adversity and infant temperament are associated with each other (Jansen et al., 2009) and temperament is influenced by environmental and well as genetic factors (Rothbart & Bates, 2006), more research is required to establish whether these factors have independent effects on self‐control or whether the associations found in the extant literature reflect more complex chains of mediating effects or confounding.

Second, evidence from interactionist models suggests that environmental and individual factors mutually and interactively shape variations in children's adjustment (Belsky & Pluess, 2009b; Boyce & Ellis, 2005; Rothbart et al., 1994). For example, traditional dual‐risk or diathesis‐stress models suggest children with certain temperamental characteristics may be more vulnerable to adverse environmental stressors (Monroe & Simons, 1991). Theories of differential susceptibility or sensitivity to context take this proposition further by suggesting that children's temperamental dispositions can both magnify and attenuate the effects of exposure to conditions of psychosocial adversity (Belsky & Pluess, 2009b). Overall, these perspectives suggest highly reactive children do worse in adverse environments and potentially better in low‐stress settings (Boyce & Ellis, 2005). Reactive temperaments have most typically (though not exclusively) been operationalized via measures of negative emotionality, including heightened distress to change and a propensity toward fussing and crying. These operationalizations relate to the factor of negative affectivity, a key dimension of Rothbart and colleagues' (1994) psychobiological model of temperament as well as a number of other models of temperament (Stifter & Dollar, 2016). The differential susceptibility framework suggests these temperamental characteristics are evolutionary markers of developmental susceptibility, and more specifically, highly reactive infants are hypothesized to possess nervous systems that are disproportionately activated in response to negative stimuli (Belsky & Pluess, 2009a).

Existing research drawing on interactionist models of child development has generally examined children's early social context by focusing on the parent–child relationship (e.g., Belsky, Hsieh, & Crnic, 1998; Belsky & Pluess, 2009b; Morris et al., 2002). While most evidence of temperament by risk interactions is from samples of middle childhood, preadolescent children, and adolescent children, there is some support for these interactionist models in relation to children's early self‐control. For instance, the association between supportive parental contexts (i.e., high mother–infant affective synchrony) and children's self‐control was found to be stronger for children identified as having “difficult” temperaments (defined as a higher propensity to display fussiness and negative emotions) compared to children with “easier” temperaments (Feldman et al., 1999). However, less work has examined how infant temperament might moderate the association between socioeconomic adversity and children's self‐control. One study that did examine this question for children's executive function at age 4 years found differential effects depending on the type of socioeconomic stressor examined (Raver et al., 2013). In support of differential susceptibility, they found infants with more reactive temperaments were more susceptible to the negative effects of subjective financial strain (compared to less reactive infants). However, they also found more reactive infants were less susceptible to the negative effects of income poverty. These findings are counterintuitive, as they suggest high reactivity is a protective factor against income poverty but a risk factor in the context of subjective experienced financial strain. Therefore, further tests of these effects are required before we can be confident in the robustness of such findings.

## The Current Study

In this article, we expand models of family stress and differential susceptibility by taking into account the complexity of socioeconomic factors shaping individual development. First, while there is robust evidence that children's self‐control is associated with family socioeconomic resources, there is as yet little understanding of whether these findings are driven primarily by objective or subjective experiences of socioeconomic adversity. Therefore, we take into account a range of objective indicators, such as parental education, employment, income, family structure, and housing conditions, as well as subjective experiences, including financial and emotional strain, and decompose their effects on early levels of self‐control. We first examine whether individual socioeconomic risk factors have unique effects on children's self‐control at age 3 years. We then test a key assumption of the family stress model, that is, whether these effects are mediated by parental experiences of subjective strain.

Second, we examine the interplay of socioeconomic risk factors and individual temperamental differences in predicting children's self‐control. Specifically, we test three hypotheses: (a) the independent effects hypothesis (i.e., socioeconomic risk and infant temperament are both expected to predict unique variance in self‐control), (b) the mediation hypothesis (i.e., associations between socioeconomic risk and self‐control are expected to be explained by infant temperament), and (c) the differential susceptibility hypothesis (i.e., associations between socioeconomic risk and self‐control vary as a function of infant temperament).

The present study extends previous research evidence in several ways: First, we assess the role of multiple socioeconomic risk factors (comprising objective as well as subjective indicators) in shaping early levels of self‐control; second, we take into account the role of infant temperament as a potential mediator and moderator of individual aspects of socioeconomic risk; third, we use evidence from a nationally representative sample.

## Method

#### Participants and Procedure

Participants are from the Millennium Cohort Study (MCS), a nationally representative longitudinal study of 18818 infants born into 18552 families in the UK during 2000 and 2001. We restricted the sample to one child per family in the 266 families containing twins and triplets. The sample was stratified to ensure adequate representation of children from the four UK countries and from disadvantaged and ethnic minority populations. Probability weights are used to account for the stratified sample design. At the first wave, data were collected via parent interviews when children were approximately 9 months old. Eighty percent of these families took part again at Wave 2 when children were approximately 3 years old (the majority of children were aged 3 months and 9 months at interview). Detailed information on the study sample and procedures has been reported elsewhere (Plewis, Calderwood, Hawkes, Hughes, & Joshi, 2007), and all data used here are available from the UK Data Service (http://www.ukdataservice.ac.uk).

#### Measures

##### Objective Socioeconomic Risk Factors

Nine indicators of socioeconomic risk were measured at Wave 1. Family income was measured as OECD equivalized monthly income (£), which is adjusted for family size and the age of family members. Parental education was measured with the highest‐level qualification of either parent on a 6‐point scale ranging from 0 (*no qualifications*) to 5 (*higher/postgraduate degree*). Occupational class was defined as the highest occupational class of either parent's current or most recent occupation using the UK's National Statistics Socioeconomic Classification, a 3‐point scale where 1 = routine and manual occupations, 2 = intermediate occupations, and 3 = higher managerial, administrative, and professional occupations. Parental unemployment was defined as families where no parent was working. Single‐parent status was assigned if only one parent lived in the household. Maternal age at the study child's birth was measured in years. Family size was measured as the number of children in the household (including the study child). Lack of home ownership was defined as any living arrangement other than parents owning their own home (e.g., renting, living with parents). Overcrowded housing was defined as living in a household containing more people than rooms suitable for sleeping (this excluded bathrooms, toilets, halls, and garages; Evans, Lercher, & Kofler, 2002).

##### Subjective Measures of Financial and Emotional Distress

Mothers' self‐reported financial distress at Wave 1 was measured with a single item that asked how well they and their partner (if applicable) were managing financially (responses ranged from 1 = *living comfortably* to 5 = *finding it very difficult*). Mothers' self‐reported psychological distress at Wave 1 was measured with nine items (α = .73) from the Rutter Malaise Inventory (Rutter, Tizard, & Whitmore, 1970). High scores on both scales indicate higher distress.

##### Self‐Control

At Wave 2, self‐control was measured by parent reports on the five‐item Hyperactivity/Inattention subscale of the Strengths and Difficulties Questionnaire (SDQ), which shows good scale reliability (α = .71) and has been widely validated as a screening tool for children's attention and hyperactivity problems (Goodman, 1997, 2001). Items of the SDQ subscale were recoded and summed so that higher scores indicate better self‐control. Though the SDQ subscale was originally developed to assess levels of self‐regulatory difficulties, it shows good face validity for measuring both the presence and absence of self‐control. This is consistent with dual‐systems models of self‐control, which suggest low self‐control can come about due to strong impulses, weak restraint, or a combination of these (Carver, 2005; Tao, Wang, Fan, & Gao, 2014). Similarly, the most widely used contemporary self‐control measure used with adults and older children, the Brief Self‐Control Scale (Tangney, Baumeister, & Boone, 2004), has been found to tap both restraint (i.e., effortful self‐control) and impulsivity domains (Maloney, Grawitch, & Barber, 2012).

The questions in the SDQ scale assess attentional control (“easily distracted, concentration wanders”), inhibitory control (“can stop and think things out before acting”). and perseverance (“sees tasks through to the end, good attention span”), as well as assessing hyperactive behavior (“restless, overactive, cannot sit still for long” and “constantly fidgeting or squirming”), which is also related to poor inhibitory control (Diamond, 2013). Attentional and inhibitory control are core components of typical definitions of self‐control and effortful control, and measures of attention (e.g., Stroop tasks) and inhibition (e.g., delay of gratification tasks) are frequently used to measure self‐control in laboratory settings (Spinrad, Eisenberg, & Gaertner, 2007). An individual's perseverance on experimental tasks is also widely used to measure self‐control (Eisenberg et al., 2009).

We tested the convergent and discriminant validity of the SDQ Hyperactivity/Inattention subscale in two ways: (a) using available data in MCS and (b) using new contemporary data collected especially for this purpose. This approach follows a number of recent studies that measure self‐control via scales originally designed to measure self‐regulation difficulties (e.g., impulsivity) in order to conduct secondary analyses of existing data sets (e.g., Daly, Delaney, Egan, & Baumeister, 2015; Moffitt et al., 2011).

Our main self‐control measure based on the SDQ was positively associated with alternative measures of self‐control collected from two independent sources in the MCS. First, our self‐control measure was positively correlated with a teacher‐rated measure of attentional control and persistence (*r* = .20, *p* < .001). Second, the survey interviewer's ratings of the study child's attentional focus during standardized vocabulary and school readiness assessments were positively associated with the SDQ‐based measure of self‐control (*r* = .18, *p* < .001). Though correlations were modest in size, we suggest this is likely due to the different raters used for each measure (the teacher rating was also conducted at a later study wave). For instance, a similar magnitude of association was shown between the teacher‐rated and interviewer‐rated measures of self‐control (*r* = .18, *p* < .001), suggesting a consistent level of cross‐rater correlation.

In the new data set we collected (*n* = 92), the SDQ Hyperactivity/Inattention subscale was positively correlated with two scales measuring effortful control selected from the short‐form Children's Behavior Questionnaire (CBQ; Rothbart, Ahadi, Hershey, & Fisher, 2001): Attentional Focusing (*r* = .44, *p* < .001) and Inhibitory Control (*r* = .60, *p* < .001). Furthermore, support for the discriminant validity of our self‐control measure was provided by results showing nonsignificant associations with two subscales of the CBQ measuring negative affect: Fear' (*r* = –.06, *p* = .56) and Sadness (*r* = –.05, *p* = .62).

Thus, both assessments provided support for the convergent and discriminant validity of the SDQ Hyperactivity/Inattention subscale with commonly used measures of childhood self‐control (more detail on these analyses is provided in Section 2 of the supplementary materials).

##### Infant Temperament

Individual differences in infant temperament were measured at Wave 1 primarily using items selected from the Carey Infant Temperament Scale (ITS; Carey & McDevitt, 1978), a widely used measure of temperament that has demonstrated good validity and reliability (Rothbart & Bates, 2006). A number of the ITS's subscales (e.g., Rhythmicity)—which are based on Chess, Thomas, and Birch's (1968) behavioral‐style model of temperament—have been dropped from contemporary temperament theories (e.g., the psychobiological model of Rothbart et al., 1994). We therefore created two measures of negative affectivity/reactivity that were closely aligned with Rothbart and colleagues' concept of negative affectivity and Belsky and colleagues' (1998) concept of reactivity, that is, measures of negative mood and withdrawal. *Negative mood* was assessed with eight items measuring the frequency with which infants cry and fuss (e.g., “How often does she become upset—by crying or screaming when she doesn't get what she wants?') and whether infants react badly to everyday events such as diaper changing and hair brushing (α = .60). This scale is related to the sadness and anger dimensions of Rothbart and colleagues' (1994) negative affectivity factor. *Withdrawal* was assessed with five items measuring infants' tendencies to withdraw and show negative affect in response to novelty (e.g., “for the first few minutes in a new place or situation she is fretful” and “is shy (turns away or clings to you) on meeting another child for the first time”; α = .67). This scale is related to the fear dimension of Rothbart and colleagues' negative affectivity factor and also Kagan's concept of behavioural inhibition (Kagan, Reznick, & Snidman, 1987; Rothbart & Bates, 2006).

##### Covariates

The child's birthweight in kilograms was reported by the main parental respondent and was used as a continuous measure. Other covariates were the child's sex (boys = 0; girls = 1) and ethnicity. Dummy variables were used to compare minority ethnic groups (Mixed = 3%; Indian = 1%; Pakistani or Bangladeshi = 2%; Black = 2%; Other = 1%) to the majority White ethnic group (91%). Minority ethnic groups were not directly compared to each other, as there are many ethnic groups in the UK and most are generally small in size. Black African and Black Caribbean were combined due to the small sample size of these groups.

#### Data Analysis

The primary analyses were a series of path models run in a format akin to hierarchical regression models where predictor variables were added to the model in four predetermined steps. Step 1 simultaneously tested the associations between all nine objective socioeconomic risk factors and self‐control. Step 2 added maternal financial and emotional distress to the model. Step 3 added the two measures of infant temperament. Step 4 added interaction terms between each of the socioeconomic risks and the two measures of infant temperament. Covariates were controlled for in all models (sex, birthweight, ethnicity). These models were fully saturated and therefore fit the data perfectly. The statistical significance of indirect effects was assessed using bootstrapped confidence intervals (Hayes & Scharkow, 2013). Maternal age and family income were rescaled (divided by 10 and 100, respectively) to prevent “ill‐scaling” (i.e., having variables with vastly different variance statistics), which can result in model convergence problems (Kline, 2011). Robustness checks supported the assumption of linear associations (e.g., testing associations held for log‐transformed predictors).

Models were run using Mplus version 7 (Muthén & Muthén, 2012), handling missingness with full‐information maximum‐likelihood (FIML) estimation (Enders, 2010). Missingness was primarily due to nonparticipation at Wave 2, resulting in 4,502 missing cases on the outcome variable (24%); all other variables had very low levels of missing data (< 4%). Attrition analyses are shown in Supplementary Table S1. A sensitivity analysis was performed where data were analyzed using listwise deletion (LD) methods (*N* = 12,811–13,028); results were almost identical using both methods (LD equivalents of the main analyses shown in Supplementary Table S5), and therefore we report the FIML‐based models due to their larger sample size (*N* = 18,552) and because the full sample is representative of the entire UK population of infants born in 2000 and 2001.

## Results

Descriptive statistics for study variables are shown in Table 1. Correlations in Table 1 show that all nine individual socioeconomic factors were correlated with children's later self‐control. The correlations between socioeconomic risk and self‐control were only small. Socioeconomic factors tended to be correlated with each other, suggesting that exposure to socioeconomic risks tends to co‐occur.

**Table 1:** FIML Estimated Correlations and Descriptive Statistics for Study Variables

		1	2	3	4	5	6	7	8	9	10	11	12	13	14	15	16
1.	Self‐control																
2.	Maternal financial stress	–.14***															
3.	Maternal emotional distress	–.18***	.25***														
4.	Negative mood	–.11***	.07***	.20***													
5.	Withdrawal	–.06***	.09***	.14***	.27***												
6.	Income	.19***	–.43***	–.15***	.02	–.11***											
7.	Education level	.22***	–.27***	–.12***	.06***	–.08***	.53***										
8.	Occupational class	.21***	–.29***	–.14***	.03**	–.09***	.56***	.61***									
9.	Unemployment	–.14***	.29***	.14***	.01	.09***	–.45***	–.44***	–.41***								
10.	Single parent	–.12***	.23***	.09***	–.01	.05***	–.36***	–.37***	–.35***	.63***							
11.	Maternal age	.18***	–.14***	–.08***	.01	–.05***	.39***	.34***	.42***	–.30***	–.28***						
12.	Family size	.03**	.13***	.06***	.00	.10***	–.20***	–.16***	–.11***	.10***	–.03**	.30***					
13.	Non‐homeowner	–.19***	.30***	.15***	.00	.09***	–.52***	–.45***	–.49***	.51***	.44***	–.43***	.07***				
14.	Overcrowding	–.05***	.13***	.08***	.03**	.09***	–.22***	–.21***	–.18***	.14***	.01	–.03**	.39***	.23***			
15.	Female gender	.13***	.00	–.02	–.03**	.06***	.00	.01	–.01	.00	–.01	.01	.01	.00	.02		
16.	Birthweight	.08***	–.05***	–.06***	.02	–.02	.09***	.12***	.10***	–.10***	–.07***	.07***	.05***	–.10***	–.04***	–.10***	
	*M*	6.12	2.23	1.62	19.05	9.85	3.27	2.90	2.13	0.17	0.14	2.89	1.90	0.36	0.09	0.49	3.37
	*SD*	2.35	1.00	1.72	4.82	3.78	2.09	1.38	0.89	0.37	0.34	0.58	1.02	0.48	0.29	0.50	0.58

*Note*. FIML = full‐information maximum‐likelihood.

*p < .05. **p < .01. ***p < .001.

#### Are Individual Indicators of Socioeconomic Risk Uniquely Associated With Children's Self‐Control?

We first tested whether individual objective socioeconomic risk factors uniquely predicted children's early self‐control after controlling for key covariates (Model 1, Table 2). Results of this analysis showed that six of the nine socioeconomic risks were uniquely associated with children's self‐control at age 3 years (*R* ^2^ = .09). Five socioeconomic risk factors were associated with lower self‐control; in order of descending (standardized) effect size, these were lower parental education (β = .10), lower parental occupational class (β = .07), lack of home ownership (β = –.06), followed by lower household income (β = .05) and younger maternal age at birth (β = .05), which showed similar effect sizes to each other.

**Table 2:** Predicting Self*‐*Control at Age 3 Years

	Model 1		Model 2		Model 3	
	*B* [95% CI]	β	*B* [95% CI]	β	*B* [95% CI]	β
Socioeconomic factors						
Income	.06 [.03, .09]	.05***	.04 [.01, .07]	.04**	.05 [.02, .08]	.04**
Parental education	.17 [.13, .22]	.10***	.17 [.13, .22]	.10***	.19 [.14, .23]	.11***
Parental occup. class	.18 [.11, .25]	.07***	.16 [.09, .23]	.06***	.17 [.10, .24]	.06***
Parental unemployment	–.07 [–.24, .11]	–.01	–.01 [–.18, .17]	.00	.01 [–.16, .18]	.00
Single parent	.06 [–.12, .24]	.01	.05 [–.12, .23]	.01	.05 [–.13, .22]	.01
Mother's age	.18 [.08, .29]	.05***	.19 [.09, .29]	.05***	.19 [.09, .29]	.05***
Family size	.12 [.06, .17]	.05***	.13 [.08, .18]	.06***	.12 [.07, .18]	.05***
Non‐homeowner	–.30 [–.42, –.17]	–.06***	–.23 [–.36, –.11]	–.05***	–.23 [–.36, –.11]	–.05***
Overcrowded housing	–.01 [–.18, .15]	.00	.01 [–.16, .19]	.00	.03 [–.14, .21]	.00
Maternal stress						
Financial stress			–.06 [–.11, –.01]	–.02*	–.05 [–.10, 00]	–.02
Emotional distress			–.19 [–.21, –.16]	–.14***	–.16 [–.19, –.13]	–.12***
Child temperament						
Negative mood					–.04 [–.05, –.03]	–.09***
Withdrawal					.00 [–.01, .01]	.00
Covariates						
Female gender	.63 [.54, .72]	.13***	.62 [.53, .70]	.13***	.61 [.52, .69]	.13***
Birthweight	.20 [.12, .28]	.05***	.18 [.10, .26]	.05***	.19 [.11, .27]	.05***
Ethnicity (ref.: White)						
Mixed	.08 [–.19, .35]	.01	.12 [–.14, .38]	.01	.16 [–.10, .42]	.01
Indian	–.26 [–.58, .06]	–.01	–.16 [–.48, .16]	–.01	–.15 [–.48, .18]	–.01
Pakistani/Bangladeshi	–.38 [–.61, –.15]	–.03**	–.31 [–.53, –.08]	–.03**	–.27 [–.50, –.04]	–.02*
Black	.29 [.01, .56]	.02*	.33 [.07, .59]	.02*	.40 [.14, .66]	.03**
Other	–.03 [–.51, .46]	.00	–.05 [–.52, .42]	.00	.03 [–.44, .50]	.00
Model *R* ^2^	9%		11%		12%	

*Note*. Model 4 consisted of Model 3 plus interaction terms; as interactions were all non–significant (*p* > .05), results for this model are shown only in Supplementary Table S4.

*p < .05. **p < .01. ***p < .001.

In contrast, large family size did not place children at risk of lower self‐control; in fact, children from larger families had higher self‐control than those from smaller families (β = .05) after controlling for exposure to the eight other socioeconomic risk factors and covariates. Parental unemployment, single parenthood, and living in overcrowded housing showed no significant association with children's level of self‐control in the multivariate model.

Male sex and lower birthweight were both associated with lower self‐control. Compared to children of White ethnicity, children of Black ethnicity scored higher on parent‐rated self‐control at age 3 years, whereas children of Pakistani or Bangladeshi ethnicity scored lower than children of White ethnicity.

#### Does Mothers' Subjective Distress Explain the Association Between Socioeconomic Risk and Lower Self‐Control in Children?

In Model 2, we added indicators of mothers' financial stress and emotional distress as predictors of children's self‐control in order to test whether they explained the associations between socioeconomic risk factors and children's self‐control (Table 2). We found that higher levels of financial stress (β = –.02) and emotional distress (β = –.14) were both associated with lower self‐control and explained a small proportion of additional variance in self‐control (Δ*R* ^2^ = .02). However, all of the associations between socioeconomic risk factors and self‐control found in Model 1 remained statistically significant with little change in effect size. In addition to their direct associations, income, occupational class, and non–home ownership had small indirect effects via mothers' feelings of financial and emotional distress, which explained between 11% and 33% of their total effect on children's self‐control (Supplementary Table S2). Tests of indirect effects (Supplementary Table S2) also showed two variables which did not have direct effects on self‐control had small negative indirect associations via mothers' financial and emotional distress, these were parental unemployment (indirect *B* = −0.06, *p* < .01) and overcrowded housing (indirect *B* = −0.02, *p* < .05). Although the direct effect of larger family size was positive, a very small negative effect via maternal stress was also observed (indirect *B* = −0.01, *p* < .01). Overall, there was evidence of only relatively weak mediated effects via mothers' financial and emotional distress, suggesting that objective socioeconomic adversity and subjective experiences of distress are largely unique predictors of children's early self‐control.

#### Interplay Between Socioeconomic Risk and Infant Temperament

Two indicators of infant temperament, negative mood and withdrawal, were added as predictors of children's self‐control in Model 3. This model showed that children rated as displaying higher levels of negative mood at age 9 months had lower self‐control at age 3 years (β = –.09) over and above indicators of objective and subjective socioeconomic risk. Infant withdrawal was not significantly associated with self‐control. Associations between self‐control and other risk factors were barely affected by the addition of these measures to the model (see Table 2). For the risk factors exhibiting direct effects in previous models, supplementary analyses indicated between 16% and 17% of their total effects were via infant temperament (Supplementary Table S3). Other negative indirect effects were via parental unemployment and overcrowding; again, these were small (Supplementary Table S3). Very little of the effects of socioeconomic risk on self‐control were explained by effects via infant temperament, providing little support for the mediation hypothesis. Overall, these findings were most consistent with the independent effects hypothesis, as socioeconomic risk and infant temperament both predicted unique variance in children's self‐control and mediated effects were very small.

In Model 4, we added 22 interaction terms to test the differential susceptibility hypothesis, that is, whether the influence of objective and subjective risk factors differed depending on individual differences in children's temperament. When all of the interaction terms were added to the model simultaneously, none of the interaction terms were statistically significant (all *p*s > .05; results shown in Supplementary Table S4). As a precaution against overfitting our model, we also tested interaction terms separately. This approach resulted in a single statistically significant interaction. This was between negative mood and household income, *B* = –.006, 95% CI [–.010, –.001], *p* = .017, where higher levels of negative mood result in weaker associations between income and self‐control, simple slope at +1SD = .035, 95% CI [.004, .067], compared to low levels of negative mood, simple slope at −1SD = .059, 95% CI [.028, .089]. Therefore, this interaction does not follow a differential susceptibility or diathesis‐stress pattern; instead, negative mood appears to act as a protective factor for children in low‐income households, supporting evidence reported by Raver et al. (2013). This interaction should be treated with caution, as it does not maintain statistical significance if we correct for the number of interactions tested (*p* = .05/22 = .002). The remaining 21 (of 22) interaction terms were nonsignificant (*p* > .05).

#### Sensitivity Analyses

As a robustness test, we ran the main analyses with an abbreviated version of the self‐control measure that excluded the two hyperactivity items. Results were broadly replicated, though the direct effects of family size and non–home ownership did not reach statistical significance (*p* > .05). Results are given in full in the appendix (Supplementary Table S5).

## Discussion

This study provides important insights into the factors that may hinder or promote the development of self‐control. It builds on a body of work that has linked lower levels of self‐control in children to contextual risk factors. More specifically, it responds to calls to disaggregate and examine the unique effects of the individual risk factors that tend to constitute cumulative risk measures and to test the potential mechanisms of these effects (Lengua et al., 2007). While a number of studies have assessed the relationship between cumulative risk and children's self‐control, there has not yet been an explicit focus on individual socioeconomic risk factors, and little is known about their unique direct effects. The current study used a hierarchical analysis to provide an assessment of the unique associations between individual socioeconomic risk factors and children's self‐control. In this study, we found unique effects of multiple socioeconomic factors on children's early self‐control. Mothers' subjective feelings of distress explained additional variance in children's self‐control but only partially accounted for the link with objective indicators of socioeconomic risk. Finally, when examining the interplay between socioeconomic risk and infant temperament, we found evidence for largely independent effects along with weak mediating effects. Very limited support for interaction effects was found.

Like previous studies, we found that both parental education and income explain unique variance in children's early self‐control (e.g., Sektnan, McClelland, Acock, & Morrison, 2010). We further extend these findings to show that additional facets of socioeconomic status (i.e., occupational class) as well as housing tenure and parental age independently predict early self‐control too. The main effects of individual risk factors on early self‐control show strong correspondence with the bivariate correlations reported by Lengua et al. (2007) using a smaller purposive sample of 3‐year‐olds (*N* = 80). For instance, Lengua and colleagues found effortful control had significant negative correlations with residential instability and income poverty. While their study did not have the statistical power available to the current study, like us, they did find a pattern of (nonsignificant) correlations indicative of younger parenthood being associated with lower self‐control. Findings related to parental unemployment were much more consistent with the family stress model, where its effects on children's early self‐control appear to be through exposure to other co‐occurring socioeconomic risks as well as via increased parental financial strain and distress (Conger et al., 2010). The current study makes the important contribution of providing evidence that parental education, income, social class, age, and housing tenure are all uniquely associated with lower self‐control in a large, representative sample of children who have not yet reached school age.

Our results support previous findings that indicate aspects of residential risk are robust predictors of low self‐control (Lengua et al., 2007; Li‐Grining, 2007). Specifically, we found children living in owner‐occupied homes at age 9 months were rated higher on self‐control than children residing in homes not owned by their parents. Home ownership has previously been shown to be associated with lower levels of emotional and behavioral problems and higher school achievement in children (Boyle, 2002). Possible processes underlying such associations include security associated with material resources and assets, the higher‐quality housing and neighborhoods accessible to homeowners, as well as (potentially pre‐existing) differences between homeowners and non‐owners on characteristics that are also beneficial to parenting, such as planning, discipline, commitment, and future orientation (Boyle, 2002; Leventhal & Newman, 2010). The latter explanation relates to a very important point about causal processes potentially underlying many of the findings presented here. It is plausible that shared genetic factors explain many of the observed associations between parental socioeconomic markers and children's self‐control. Two types of studies could provide further insight into this issue: (a) genetically informed designs such as twin or adoption studies and (b) statistical methods such as fixed effects models that explore longitudinal change in both parental SES and children's self‐control while controlling for time‐invariant covariates. This is an important area for future research.

We also examined residential overcrowding in the current study, as previous research has linked living in overcrowded homes to adjustment difficulties in children, including teacher‐rated behavioral problems and grade retention (Leventhal & Newman, 2010). We did not find a direct association between early self‐control and overcrowding, though some small indirect effects were found via mothers' experiences of distress and infant temperament. One possible reason for only finding modest indirect associations in the current study could be due to our focus very early on in childhood. For instance, a key drawback of living in overcrowded housing is the lack of personal space and privacy it provides (Leventhal & Newman, 2010); however, this is unlikely to be as important an issue for infants as it would be for older children and adolescents.

Compared to indicators of socioeconomic status, maternal age seems to have less influence on aspects of children's behavioral regulation, such as externalizing problems (Deater‐Deckard, Dodge, Bates, & Pettit, 1998). While a number of studies have included adolescent parenthood in their cumulative risk measures (Lengua et al., 2007, 2015; Li‐Grining, 2007), robust tests of the unique association between parental age and children's early self‐control have been lacking. Nevertheless, in the current study, we found that children born to younger mothers had lower parent‐reported self‐control at age 3 years. These findings are comparable to existing data that show a general trend of young parenthood being associated with lower self‐control in early childhood, but it is important to note that this is largely based on zero‐order correlations from smaller samples, which have not always reached conventional significance thresholds (Lengua et al., 2007, 2015). Overall, findings point toward an association between maternal age and children's self‐control, yet more research is required that combines the sample size strengths of the current study with the robust, objective measurement strengths of previous studies.

Although we found single‐parent status was correlated with higher maternal distress and lower self‐control, our findings point to virtually no unique associations between these variables after we control for other aspects of socioeconomic risk. Similar to our findings on unemployment, a key mechanism of risk associated with single parenthood is the lower family income experienced by single parents (Musick & Meier, 2010). Single parents are much more likely to have low incomes (and also be in workless households), as shown in the substantial correlations found between single‐parent status with low income and unemployment in the current study. Overall, our findings correspond to previous conclusions, where it seems that the observed bivariate correlations found between single‐parent status and lower child self‐control are due to confounding with other socioeconomic risks such as low income (Zalewski et al., 2012).

Large family size is a well‐established risk factor for conduct problems in children (Collishaw, Goodman, Pickles, & Maughan, 2007); however, we found no evidence of a negative association with children's self‐control. In fact, our findings suggest that large families (measured by the number of children in the household) tend to have more self‐controlled children. Due to the young age at which family size was assessed, nearly all siblings were older than the study child; therefore, these findings point to older siblings having a potential role in helping young children to develop self‐control. This is consistent with previous research that has shown sibling characteristics such as affection and hostility are correlated (positively and negatively, respectively) with children's self‐regulation during early adolescence (Padilla Walker, Harper, & Jensen, 2010). In sum, it seems likely that older siblings have a role that may complement the well‐established effects of parents on children's self‐control.

This study drew on a large, representative sample and is one of the first to examine the early precursors of self‐control in a UK sample. It is thus worthwhile to comment on the pattern of associations relating to our selected covariates. First, we replicate findings from other countries where girls are rated as having better self‐control than boys (Kochanska & Knaack, 2003; Li‐Grining, 2007) and also that low‐birthweight children exhibit lower levels of self‐control than their typically developing peers (Li‐Grining, 2007; Taylor, Klein, & Hack, 2000). However, we also found differences between ethnic groups that were more complex and robust than those suggested by research with predominantly North American samples. Children of Pakistani or Bangladeshi ethnicity were rated as having lower levels of self‐control compared to White ethnicity children, whereas children of Black ethnicity were rated as having higher levels of self‐control by their parents. These differences were found after controlling for socioeconomic risk and therefore do not seem to follow the same pattern found in earlier studies where lower levels of self‐control among ethnic minority children tend to be explained by the relative social disadvantage of minority ethnic groups in the United States (Piotrowski, Lapierre, & Linebarger, 2013). This pattern of differences across ethnicities is interesting but requires further verification with more objective measures of self‐control before we can be confident that they are not simply due to cultural differences in the level of self‐control expected of young children.

The family stress model suggests that parents' subjective feelings of distress mediate the effects of socioeconomic risk on children's development (Conger et al., 2010; Leventhal & Newman, 2010). To our knowledge, this study was the first to test the family stress model in relation to children's self‐control. There was some support for this model, with associations for two risk factors—unemployment and overcrowding—only occurring via mothers' subjective distress, and partial mediation found for three other objective risk factors (income, occupational class, home ownership). Nevertheless, the direct association between risk factors and self‐control was strongest, and there appears to be only a limited influence via mothers' financial and emotional distress. These results concur with previous research that has shown that the family stress model does not entirely account for the associations between ecological risk and children's outcomes (Kiernan & Huerta, 2008; Mistry et al., 2010; Schoon, Hope, Ross, & Duckworth, 2010). Further research is required to more fully explain these links; some discussion on this is provided below.

Existing developmental theories suggest that competencies such as self‐control arise from the interplay of both contextual and individual characteristics, yet to date it has remained unclear how aspects of socioeconomic risk and infants' temperamental reactivity might work together to influence the emergence of children's early self‐control. Our findings indicate roles for both social causation and social selection in the etiology of early self‐control, where the negative effects of socioeconomic risk and temperament operate simultaneously to result in lower self‐control at age 3 years. This pattern of findings is consistent with cumulative risk perspectives (Evans, Li, & Whipple, 2013), where the children with the lowest levels of self‐control are likely to be those who are experiencing the greatest number of risk factors. While a number of theoretical perspectives emphasize that developmental outcomes arise from the interaction of contextual and individual characteristics, we found little robust evidence of such effects in the current study. Theoretical work and empirical evidence on differential susceptibility focus almost exclusively on parenting (Belsky & Pluess, 2009b; Bradley & Corwyn, 2008; Hartman & Belsky, 2015); therefore, one explanation for our null findings could be that interactive effects are limited to these more proximal ecological risks rather than distal socioeconomic risks such as those studied here (Bronfenbrenner & Morris, 2006). Nevertheless, to our knowledge, the only other study of children's early self‐regulation (i.e., executive functioning at age 4 years) that explicitly tested for interactive effects between socioeconomic risk and infant temperament reported quite mixed results. We partially replicated findings by Raver et al. (2013) suggesting that reactive temperament protected slightly against income poverty, but we did not replicate their finding whereby highly reactive temperaments exacerbated the risk of perceived hardship. In light of our null findings for most of the socioeconomic risks, including objective and perceived economic stress, further evidence is required before we can be confident that such interactive effects exist at the UK population level.

It is worth noting that a relatively low proportion of variance in children's early self‐control was explained by the predictors examined here (up to 12%). This may not be particularly surprising given that we assessed socioeconomic risks at only one point in time and tested longitudinal associations lagged over a 3‐year period. The results of previous studies indicate that measuring socioeconomic risk over time (e.g., chronicity of exposure) explains more variance in self‐regulatory outcomes than baseline measures of risk alone (Raver et al., 2013). The effect sizes we reported for individual socioeconomic factors were at the smaller end of effect sizes commonly observed for variables such as gender (ranging from .12 to .23) and aspects of parenting (ranging from .09 to .34; Kochanska & Knaack, 2003; Lengua et al., 2007; Li‐Grining, 2007; Piotrowski et al., 2013). Parenting characteristics are likely to be particularly important factors that will explain additional variance in children's self‐control, including parenting behaviors aimed at reducing children's negative affect, parenting that promotes children's agency and mastery, and the provision of clear and consistent guidelines about the behaviors expected of children (Lengua et al., 2007; Ng‐Knight et al., 2016). These processes should be examined in more detail in future studies.

## Limitations

There are some limitations to bear in mind when interpreting the results of this study, most of which arise due to the nature of performing secondary analysis on existing data from a large‐scale study, therefore restricting analyses to the available data. However, despite its inherent limitations, it should be noted that utilizing existing data sources to assess the antecedents of conscientiousness traits such as self‐control has been highlighted as an important research aim (Eisenberg et al., 2014). For instance, the measures of maternal strain used may not have fully captured the stressors mothers experience as a result of socioeconomic adversity; neither was interparental conflict assessed. Future research may reduce measurement error and benefit from drawing on multi‐item measures of strain reported by both parents (e.g., Simons et al., 2016). We expect the modest indirect effects reported via maternal strain to increase in magnitude if additional aspects of family and parental functioning are included. In particular, parenting has been linked to self‐control in multiple samples with effect sizes similar to or in excess of the direct effects found here (Choe et al., 2013; Merz et al., 2015), though this has generally not been in a family stress model framework and therefore not always controlled for socioeconomic factors. However, some studies, such as Piotrowski and colleagues' (2013) cross‐sectional survey of parents of children aged 8 months up to 7 years, found parenting characteristics explained approximately 10% of the variance in children's self‐control over and above indicators of SES.

We were limited to only having parent reports of children's self‐control at age 3 years; this contrasts with smaller studies, which are often able to collect more objective, task‐based measures of attention and inhibitory control (Lengua et al., 2007, 2015; Li‐Grining, 2007). We did use a well‐validated measure of children's self‐regulatory difficulties (Goodman, 2001) and provided some preliminary validation data, but replication of the associations presented here using alternative measurement methods would build confidence in the findings. This study measured infant temperament in line with differential susceptibility theory's concept of reactivity, but future research may benefit from assessing additional aspects of temperament, such as attentional focus, that are key to contemporary theories of temperament (Rothbart & Bates, 2006).

Our inclusion of risk factors was largely guided by existing research linking cumulative ecological risks to children's early self‐control (Lengua et al., 2007, 2015; Li‐Grining, 2007); therefore, we may have omitted other important variables from the model that could have altered the results (e.g., neighborhood risk, housing quality). There was some attrition between study waves, which can lead to biased estimates; however, we found very similar results when using both complete‐case and FIML methods, which provides some confidence in the robustness of the associations found here. Finally, the non‐experimental design used here cannot directly assess questions of causality. We would also like to emphasize the importance of testing whether the observed associations between SES and self‐control are genetically or environmentally mediated.

## Conclusions

In a large, nationally representative sample, infants living in families characterized by lower income, lower parental education, lower occupational class, a lack of home ownership, and younger mothers had lower self‐control at age 3 years. There were independent effects of socioeconomic risks, mothers' subjective distress, and infant temperament, as well as indirect effects of socioeconomic risks via maternal distress and infant temperament. Taken together, the findings support a bioecological model of development, rather than a differential susceptibility model, where self‐control is influenced by characteristics of the individual and their social context as well as the reciprocal relations between these factors (Bronfenbrenner & Morris, 2006). Given the broad developmental implications of low self‐control, which span mental, physical, and financial health, our results lend support to the potential benefits of alleviating socioeconomic and residential disadvantage in families with infants and young children.

### Declaration of Conflicting Interests

The authors declared no potential conflicts of interest with respect to the research, authorship, and/or publication of this article.

### Funding

The authors disclosed receipt of the following financial support for the research, authorship, and/or publication of this article: Preparation of this manuscript was supported by a postdoctoral Fellowship award to Terry Ng‐Knight from the Jacobs Foundation as well as funding for Ingrid Schoon from the Wissenschaftszentrum Berlin (WZB), and Grant Number ES/J019658/1 and ES/L008211/1 from the British Economic and Social Research Council (ESRC).

## Supporting information


**Supplementary table S1**. Results of attrition analyses showing sweep 1 indicators of non‐participation at sweep 2.
**Supplementary table S2**. Indirect effects of socioeconomic factors on self‐control via maternal stress
**Supplementary table S3**. Indirect effects of socioeconomic factors on self‐control via infant temperament
**Supplementary table S4**. Model 4 containing interaction terms.
**Supplementary Table S5**. Results of main analyses conducted on complete cases (listwise deletion)
**Supplementary Table S6**. Results of main analyses conducted with abbreviated self‐control measure.

## References

[jopy12288-bib-0001] Belsky, J. , Hsieh, K.‐H. , & Crnic, K. (1998). Mothering, fathering, and infant negativity as antecedents of boys' externalizing problems and inhibition at age 3 years: Differential susceptibility to rearing experience? Development and Psychopathology, 10, 301–319. 9635226 10.1017/s095457949800162x

[jopy12288-bib-0002] Belsky, J. , & Pluess, M. (2009a). Beyond diathesis stress: Differential susceptibility to environmental influences. Psychological Bulletin, 135, 885–908. 19883141 10.1037/a0017376

[jopy12288-bib-0003] Belsky, J. , & Pluess, M. (2009b). The nature (and nurture?) of plasticity in early human development. Perspectives on Psychological Science, 4, 345–351. 26158982 10.1111/j.1745-6924.2009.01136.x

[jopy12288-bib-0004] Blair, C. (2010). Stress and the development of self‐regulation in context. Child Development Perspectives, 4, 181–188. 21779305 10.1111/j.1750-8606.2010.00145.x PMC3138186

[jopy12288-bib-0005] Boyce, W. T. , & Ellis, B. J. (2005). Biological sensitivity to context: I. An evolutionary–developmental theory of the origins and functions of stress reactivity. Development and Psychopathology, 17, 271–301. 16761546 10.1017/s0954579405050145

[jopy12288-bib-0006] Boyle, M. H. (2002). Home ownership and the emotional and behavioral problems of children and youth. Child Development, 73, 883–892. 12038558 10.1111/1467-8624.00445

[jopy12288-bib-0007] Bradley, R. H. , & Corwyn, R. F. (2008). Infant temperament, parenting, and externalizing behavior in first grade: A test of the differential susceptibility hypothesis. Journal of Child Psychology and Psychiatry, 49, 124–131. 18211274 10.1111/j.1469-7610.2007.01829.x

[jopy12288-bib-0008] Bronfenbrenner, U. , & Morris, P. A. (2006). The bioecological model of human development In Lerner R. M. (Ed.), Handbook of child psychology: Theoretical models of human development (6th ed., Vol. 1, pp. 793–828). Hoboken, NJ: Wiley.

[jopy12288-bib-0009] Carey, W. B. , & McDevitt, S. C. (1978). Revision of the Infant Temperament Questionnaire. Pediatrics, 61, 735–739. 662513

[jopy12288-bib-0010] Carlson, S. M. , Mandell, D. J. , & Williams, L. (2004). Executive function and theory of mind: Stability and prediction from ages 2 to 3. Developmental Psychology, 40, 1105–1122. 15535760 10.1037/0012-1649.40.6.1105

[jopy12288-bib-0011] Carver, C. S. (2005). Impulse and constraint: Perspectives from personality psychology, convergence with theory in other areas, and potential for integration. Personality and Social Psychology Review, 9, 312–333. 16223354 10.1207/s15327957pspr0904_2

[jopy12288-bib-0012] Caspi, A. , Roberts, B. W. , & Shiner, R. L. (2005). Personality development: Stability and change. Annual Review of Psychology, 56, 453–484. 10.1146/annurev.psych.55.090902.141913 15709943

[jopy12288-bib-0013] Chess, S. , Thomas, A. , & Birch, H. (1968). Temperament and behavior disorders in children. New York: New York University Press.

[jopy12288-bib-0014] Choe, D. E. , Olson, S. L. , & Sameroff, A. J. (2013). Effects of early maternal distress and parenting on the development of children's self‐regulation and externalizing behavior. Development and Psychopathology, 25, 437–453. 23627955 10.1017/S0954579412001162

[jopy12288-bib-0015] Collishaw, S. , Goodman, R. , Pickles, A. , & Maughan, B. (2007). Modelling the contribution of changes in family life to time trends in adolescent conduct problems. Social Science & Medicine, 65, 2576–2587. 17881107 10.1016/j.socscimed.2007.06.010

[jopy12288-bib-0016] Conger, R. D. , Conger, K. J. , & Martin, M. J. (2010). Socioeconomic status, family processes, and individual development. Journal of Marriage and the Family, 72, 685–704. 20676350 10.1111/j.1741-3737.2010.00725.x PMC2910915

[jopy12288-bib-0017] Daly, M. , Delaney, L. , Egan, M. , & Baumeister, R. F. (2015). Childhood self‐control and unemployment throughout the life span: Evidence from two British cohort studies. Psychological Science, 26, 709–723. 25870404 10.1177/0956797615569001 PMC4512256

[jopy12288-bib-0018] Deater‐Deckard, K. , Dodge, K. A. , Bates, J. E. , & Pettit, G. S. (1998). Multiple risk factors in the development of externalizing behavior problems: Group and individual differences. Development and Psychopathology, 10, 469–493. 9741678 10.1017/s0954579498001709 PMC2776047

[jopy12288-bib-0019] Diamond, A. (2013). Executive functions. Annual Review of Psychology, 64, 135–168. 10.1146/annurev-psych-113011-143750 PMC4084861 23020641

[jopy12288-bib-0020] Eisenberg, N. , Duckworth, A. L. , Spinrad, T. L. , & Valiente, C. (2014). Conscientiousness: Origins in childhood? Developmental Psychology, 50, 1331–1349. 23244405 10.1037/a0030977 PMC3610789

[jopy12288-bib-0021] Eisenberg, N. , Valiente, C. , Spinrad, T. L. , Cumberland, A. , Liew, J. , Reiser, M. , et al. (2009). Longitudinal relations of children's effortful control, impulsivity, and negative emotionality to their externalizing, internalizing, and co‐occurring behavior problems. Developmental Psychology, 45, 988–1008. 19586175 10.1037/a0016213 PMC2775424

[jopy12288-bib-0022] Eisenberg, N. , Zhou, Q. , Spinrad, T. L. , Valiente, C. , Fabes, R. A. , & Liew, J. (2005). Relations among positive parenting, children's effortful control, and externalizing problems: A three wave longitudinal study. Child Development, 76, 1055–1071. 16150002 10.1111/j.1467-8624.2005.00897.x PMC1351058

[jopy12288-bib-0023] Enders, C. K. (2010). Applied missing data analysis. New York: Guilford Press.

[jopy12288-bib-0024] Evans, G. , Li, D. , & Whipple, S. (2013). Cumulative risk and child development. Psychological Bulletin, 139, 1342–1396. 23566018 10.1037/a0031808

[jopy12288-bib-0025] Evans, G. W. (2006). Child development and the physical environment. Annual Review of Psychology, 57, 423–451. 10.1146/annurev.psych.57.102904.190057 16318602

[jopy12288-bib-0026] Evans, G. W. , Lercher, P. , & Kofler, W. W. (2002). Crowding and children's mental health: The role of house type. Journal of Environmental Psychology, 22, 221–231.

[jopy12288-bib-0027] Evans, G. W. , & Schamberg, M. A. (2009). Childhood poverty, chronic stress, and adult working memory. Proceedings of the National Academy of Sciences, 106, 6545–6549. 10.1073/pnas.0811910106 PMC2662958 19332779

[jopy12288-bib-0028] Feldman, R. , Greenbaum, C. W. , & Yirmiya, N. (1999). Mother–infant affect synchrony as an antecedent of the emergence of self‐control. Developmental Psychology, 35, 223–231. 9923477 10.1037//0012-1649.35.1.223

[jopy12288-bib-0029] Goodman, R. (1997). The Strengths and Difficulties Questionnaire: A research note. Journal of Child Psychology and Psychiatry, 38, 581–586. 9255702 10.1111/j.1469-7610.1997.tb01545.x

[jopy12288-bib-0030] Goodman, R. (2001). Psychometric properties of the Strengths and Difficulties Questionnaire. Journal of the American Academy of Child and Adolescent Psychiatry, 40, 1337–1345. 11699809 10.1097/00004583-200111000-00015

[jopy12288-bib-0031] Hartman, S. , & Belsky, J. (2015). An evolutionary perspective on family studies: Differential susceptibility to environmental influences. *Family Process*. Advance online publication. doi:10.1111/famp.12161 26133233

[jopy12288-bib-0032] Hayes, A. F. , & Scharkow, M. (2013). The relative trustworthiness of inferential tests of the indirect effect in statistical mediation analysis: Does method really matter? Psychological Science, 24, 1918–1927. 23955356 10.1177/0956797613480187

[jopy12288-bib-0033] Jansen, P. W. , Raat, H. , Mackenbach, J. P. , Jaddoe, V. W. , Hofman, A. , Verhulst, F. C. , & Tiemeier, H. (2009). Socioeconomic inequalities in infant temperament. Social Psychiatry and Psychiatric Epidemiology, 44, 87–95. 18663396 10.1007/s00127-008-0416-z

[jopy12288-bib-0034] Jones, L. B. , Rothbart, M. K. , & Posner, M. I. (2003). Development of executive attention in preschool children. Developmental Science, 6, 498–504.

[jopy12288-bib-0035] Kagan, J. , Reznick, J. S. , & Snidman, N. (1987). The physiology and psychology of behavioral inhibition in children. Child Development, 58, 1459–1473. 3691195

[jopy12288-bib-0036] Kiernan, K. E. , & Huerta, M. C. (2008). Economic deprivation, maternal depression, parenting and children's cognitive and emotional development in early childhood. British Journal of Sociology, 59, 783–806. 19035922 10.1111/j.1468-4446.2008.00219.x

[jopy12288-bib-0037] Kiff, C. J. , Lengua, L. J. , & Zalewski, M. (2011). Nature and nurturing: Parenting in the context of child temperament. Clinical Child and Family Psychology Review, 14, 251–301. 21461681 10.1007/s10567-011-0093-4 PMC3163750

[jopy12288-bib-0038] Kline, R. B. (2011). Principles and practice of structural equation modeling. New York: Guilford Press.

[jopy12288-bib-0039] Kochanska, G. , & Knaack, A. (2003). Effortful control as a personality characteristic of young children: Antecedents, correlates, and consequences. Journal of Personality, 71, 1087–1112. 14633059 10.1111/1467-6494.7106008

[jopy12288-bib-0040] Kochanska, G. , Murray, K. T. , & Harlan, E. T. (2000). Effortful control in early childhood: Continuity and change, antecedents, and implications for social development. Developmental Psychology, 36, 220–232. 10749079

[jopy12288-bib-0041] Kopp, C. B. (1982). Antecedents of self‐regulation: A developmental perspective. Developmental Psychology, 18, 199–214.

[jopy12288-bib-0042] Lengua, L. (2009). Effortful control in the context of socioeconomic and psychosocial risk. Psychological Science Agenda, 23.

[jopy12288-bib-0043] Lengua, L. J. , Honorado, E. , & Bush, N. R. (2007). Contextual risk and parenting as predictors of effortful control and social competence in preschool children. Journal of Applied Developmental Psychology, 28, 40–55. 21687825 10.1016/j.appdev.2006.10.001 PMC3115727

[jopy12288-bib-0044] Lengua, L. J. , Moran, L. , Zalewski, M. , Ruberry, E. , Kiff, C. , & Thompson, S. (2015). Relations of growth in effortful control to family income, cumulative risk, and adjustment in preschool‐age children. Journal of Abnormal Child Psychology, 43, 705–720. 25253079 10.1007/s10802-014-9941-2 PMC4375071

[jopy12288-bib-0045] Leventhal, T. , & Newman, S. (2010). Housing and child development. Children and Youth Services Review, 32, 1165–1174.

[jopy12288-bib-0046] Li‐Grining, C. P. (2007). Effortful control among low‐income preschoolers in three cities: Stability, change, and individual differences. Developmental Psychology, 43, 208–221. 17201520 10.1037/0012-1649.43.1.208

[jopy12288-bib-0047] Maloney, P. W. , Grawitch, M. J. , & Barber, L. K. (2012). The multi‐factor structure of the Brief Self‐Control Scale: Discriminant validity of restraint and impulsivity. Journal of Research in Personality, 46, 111–115.

[jopy12288-bib-0048] McLoyd, V. C. (1998). Socioeconomic disadvantage and child development. American Psychologist, 53, 185–204. 9491747 10.1037//0003-066x.53.2.185

[jopy12288-bib-0049] Merz, E. C. , Landry, S. H. , Zucker, T. A. , Barnes, M. A. , Assel, M. , Taylor, H. B. , et al. (2015). Parenting predictors of delay inhibition in socioeconomically disadvantaged preschoolers. Infant and Child Development, 132, 14–31. 10.1002/icd.1946 PMC5098809 27833461

[jopy12288-bib-0050] Mistry, R. S. , Benner, A. D. , Biesanz, J. C. , Clark, S. L. , & Howes, C. (2010). Family and social risk, and parental investments during the early childhood years as predictors of low‐income children's school readiness outcomes. Early Childhood Research Quarterly, 25, 432–449.

[jopy12288-bib-0051] Moffitt, T. E. , Arseneault, L. , Belsky, D. , Dickson, N. , Hancox, R. J. , Harrington, H. , et al. (2011). A gradient of childhood self‐control predicts health, wealth, and public safety. Proceedings of the National Academy of Sciences, 108, 2693–2698. 10.1073/pnas.1010076108 PMC3041102 21262822

[jopy12288-bib-0052] Monroe, S. M. , & Simons, A. D. (1991). Diathesis‐stress theories in the context of life stress research: Implications for the depressive disorders. Psychological Bulletin, 110, 406–425. 1758917 10.1037/0033-2909.110.3.406

[jopy12288-bib-0053] Morris, A. S. , Silk, J. S. , Steinberg, L. , Sessa, F. M. , Avenevoli, S. , & Essex, M. J. (2002). Temperamental vulnerability and negative parenting as interacting predictors of child adjustment. Journal of Marriage and Family, 64, 461–471.

[jopy12288-bib-0054] Musick, K. , & Meier, A. (2010). Are both parents always better than one? Parental conflict and young adult well‐being. Social Science Research, 39, 814–830. 20824195 10.1016/j.ssresearch.2010.03.002 PMC2930824

[jopy12288-bib-0055] Muthén, L. K. , & Muthén, B. O. (2012). Mplus user's guide. Los Angeles: Muthén and Muthén.

[jopy12288-bib-0056] Newland, R. P. , Crnic, K. A. , Cox, M. J. , & Mills‐Koonce, W. R. (2013). The family model stress and maternal psychological symptoms: Mediated pathways from economic hardship to parenting. Journal of Family Psychology, 27, 96–105. 23421837 10.1037/a0031112 PMC8011847

[jopy12288-bib-0057] Ng‐Knight, T. , Shelton, K. H. , Riglin, L. , Mcmanus, I. C. , Frederickson, N. , & Rice, F. (2016). A longitudinal study of self‐control at the transition to secondary school: Considering the role of pubertal status and parenting. Journal of Adolescence, 50, 44–55. 27183536 10.1016/j.adolescence.2016.04.006

[jopy12288-bib-0058] Padilla Walker, L. , Harper, J. , & Jensen, A. (2010). Self‐regulation as a mediator between sibling relationship quality and early adolescents' positive and negative outcomes. Journal of Family Psychology, 24, 419–428. 20731488 10.1037/a0020387

[jopy12288-bib-0059] Piotrowski, J. T. , Lapierre, M. A. , & Linebarger, D. L. (2013). Investigating correlates of self‐regulation in early childhood with a representative sample of English‐speaking American families. Journal of Child and Family Studies, 22, 423–436. 23525149 10.1007/s10826-012-9595-z PMC3602616

[jopy12288-bib-0060] Plewis, I. , Calderwood, L. , Hawkes, D. , Hughes, G. , & Joshi, H. (2007). Millennium cohort study: Technical report on sampling. London: Centre for Longitudinal Studies, Institute of Education.

[jopy12288-bib-0061] Raver, C. C. , Blair, C. , & Willoughby, M. (2013). Poverty as a predictor of 4‐year‐olds' executive function: New perspectives on models of differential susceptibility. Developmental Psychology, 49, 292–304. 22563675 10.1037/a0028343 PMC5460626

[jopy12288-bib-0062] Rothbart, M. , Derryberry, D. , & Posner, M. (1994). A psychobiological approach to the development of temperament In Bates J. & Wachs T. (Eds.), Temperament: Individual differences at the interface of biology and behavior (pp. 83–116). Washington, DC: American Psychological Association.

[jopy12288-bib-0063] Rothbart, M. K. , Ahadi, S. A. , Hershey, K. L. , & Fisher, P. (2001). Investigations of temperament at three to seven years: The Children's Behavior Questionnaire. Child Development, 72, 1394–1408. 11699677 10.1111/1467-8624.00355

[jopy12288-bib-0064] Rothbart, M. K. , & Bates, J. (2006). Temperament In Eisenberg N., Damon W., & Lerner R. (Eds.), Handbook of child psychology: Social, emotional and personality development (6th ed., Vol. 3, pp. 99–166). New York: Wiley.

[jopy12288-bib-0065] Rutter, M. , Tizard, J. , & Whitmore, K. (1970). Education, health and behaviour. London: Longmans.

[jopy12288-bib-0066] Sameroff, A. (2009). The transactional model. Washington, DC: American Psychological Association.

[jopy12288-bib-0067] Schoon, I. , Hope, S. , Ross, A. , & Duckworth, K. (2010). Family hardship and children's development: The early years. Longitudinal and Life Course Studies, 1, 209–222.

[jopy12288-bib-0068] Schoon, I. , Sacker, A. , & Bartley, M. (2003). Socio‐economic adversity and psychosocial adjustment: A developmental‐contextual perspective. Social Science & Medicine, 57, 1001–1015. 12878101 10.1016/s0277-9536(02)00475-6

[jopy12288-bib-0069] Sektnan, M. , McClelland, M. M. , Acock, A. , & Morrison, F. J. (2010). Relations between early family risk, children's behavioral regulation, and academic achievement. Early Childhood Research Quarterly, 25, 464–479. 20953343 10.1016/j.ecresq.2010.02.005 PMC2953426

[jopy12288-bib-0070] Simons, L. G. , Wickrama, K. A. S. , Lee, T. K. , Landers‐Potts, M. , Cutrona, C. , & Conger, R. D. (2016). Testing family stress and family investment explanations for conduct problems among African American adolescents. Journal of Marriage and Family, 78, 498–515.

[jopy12288-bib-0071] Spinrad, T. L. , Eisenberg, N. , & Gaertner, B. M. (2007). Measures of effortful regulation for young children. Infant Mental Health Journal, 28, 606–626. 18066395 10.1002/imhj.20156 PMC2121588

[jopy12288-bib-0072] Stifter, C. , & Dollar, J. (2016). Temperament and developmental psychopathology In Cicchetti D. (Ed.), Developmental psychopathology (3rd ed., Vol. 4, pp. 546–607). Hoboken, NJ: John Wiley & Sons.

[jopy12288-bib-0073] Tangney, J. P. , Baumeister, R. F. , & Boone, A. L. (2004). High self‐control predicts good adjustment, less pathology, better grades, and interpersonal success. Journal of Personality, 72, 271–324. 15016066 10.1111/j.0022-3506.2004.00263.x

[jopy12288-bib-0074] Tao, T. , Wang, L. , Fan, C. , & Gao, W. (2014). Development of self‐control in children aged 3 to 9 years: Perspective from a dual‐systems model. Scientific Reports, 4, 7272 http://www.nature.com/articles/srep07272. 25501669 10.1038/srep07272 PMC5377018

[jopy12288-bib-0075] Taylor, H. G. , Klein, N. , & Hack, M. (2000). School‐age consequences of birth weight less than 750g: A review and update. Developmental Neuropsychology, 17, 289–321. 11056846 10.1207/S15326942DN1703_2

[jopy12288-bib-0076] Zalewski, M. , Lengua, L. J. , Fisher, P. A. , Trancik, A. , Bush, N. R. , & Meltzoff, A. N. (2012). Poverty and single parenting: Relations with preschoolers' cortisol and effortful control. Infant and Child Development, 21, 537–554.

[jopy12288-bib-0077] Zhou, Q. , Chen, S. H. , & Main, A. (2012). Commonalities and differences in the research on children's effortful control and executive function: A call for an integrated model of self‐regulation. Child Development Perspectives, 6, 112–121.

