# Supplementary material for: Disentangling the Influence of Socioeconomic Risks on Children's Early Self‐Control
Source: J Pers. 2016 Dec 10;85(6):793–806. doi: 10.1111/jopy.12288 (PMC6849748; doi:10.1111/jopy.12288)
Supplement: Supplementary file 1 — Supplementary table S1. Results of attrition analyses showing sweep 1 indicators of non‐participation at sweep 2. Supplementary table S2. Indirect effects of socioeconomic factors on self‐control via maternal stress Supplementary table S3. Indirect effects of socioeconomic factors on self‐control via infant temperament Supplementary table S4. Model 4 containing interaction terms. Supplementary Table S5. Results of main analyses conducted on complete cases (listwise deletion) Supplementary Table S6. Results of main analyses conducted with abbreviated self‐control measure. [file JOPY-85-793-s001.docx]

Supplementary Information for ‘Disentangling the influence of socioeconomic risks on children’s early self-control’.

**Authors and affiliations:**

Terry Ng-Knight ^1*^ and Ingrid Schoon ^1^

^1^ Department of Social Science, University College London Institute of Education.

^*^ Corresponding author. Department of Social Science, UCL Institute of Education, 20 Bedford Way, London, WC1H OAL.

Contact: [terry.ng-knight@ucl.ac.uk](mailto:terry.ng-knight@ucl.ac.uk)

**Section 1: Additional sample information.**

**Section 2: Validating the SDQ hyperactivity/inattention scale.**

**Section 3: Results of interaction tests.**

**Section 4: Results of sensitivity analyses.**

**Section 1: Additional sample information.**

| Supplementary table S1.  *Results of attrition analyses showing sweep1 indicators of non-participation at sweep 2.* | | | |
| --- | --- | --- | --- |
|  | Participants | Non-participants |  |
|  |  |  |  |
| *Continuous variables* | M [95% CI] | M [95% CI] | Cohen’s d |
| Income | 301.58 [298.35, 304.82] | 235.99 [230.39, 241.59] | .34 |
| Highest qualification | 2.80 [2.79, 2.83] | 2.31 [2.26, 2.36] | .35 |
| Occupational class | 2.09 [2.07, 2.10] | 1.80 [1.77, 1.83] | .33 |
| Mother’s age at child’s birth | 28.70 [28.60, 28.79] | 26.83 [26.64, 27.03] | .32 |
| Family size (number of children) | 1.95 [1.93, 1.97] | 1.96 [1.92, 1.99] | -.01 |
| Financial stress | 2.27 [2.25, 2.28] | 2.42 [2.39, 2.46] | -.16 |
| Emotional distress | 1.68 [1.65, 1.71] | 1.81 [1.75, 1.88] | -.08 |
| Infant negative mood | 19.10 [19.02, 19.18] | 18.95 [18.79, 19.12] | .03 |
| Infant withdrawal | 9.94 [9.88, 10.01] | 10.29 [10.15, 10.43] | -.09 |
| Birthweight | 3.36 [3.35, 3.36] | 3.30 [3.28, 3.32] | .10 |
| *Binary variables* | % | % | Odds Ratio |
| Parental unemployment | 18.82 | 31.94 | 2.02^***^ |
| Single parent | 15.16 | 25.37 | 1.90^***^ |
| Lack of home ownership | 37.88 | 56.94 | 2.17^***^ |
| Overcrowded housing | 11.04 | 15.63 | 1.49^***^ |
| *Note*. ^***^ = *p* *<*.001 |  |  |  |

**Section 2: Validating the SDQ hyperactivity/inattention scale.**

Following recent research into self-control based on secondary analyses of existing large-scale datasets (Daly, Delaney, Egan, & Baumeister, 2015), we used data from two sources to validate our measure of self-control. First, we used other available measures in the MCS dataset, and then, second, we collected additional contemporary data specifically for validation purposes.

**Convergent validity in the MCS**

Two alternative measures of self-control were constructed from two independent sources, using evidence collected in the MCS. The first measure consisted of two teacher-rated items assessing children’s attentional control (i.e., ‘maintains attention and concentrates’) and persistence (i.e., ‘sustains involvement and perseveres, particularly when trying to solve a problem’). This data was collected only from children in Scotland, Wales and Northern Ireland (i.e., excluded England) at a later survey sweep (i.e., sweep 3, two years after our main self-control measure was collected). Despite the time lag, this scale demonstrated convergent validity with our main self-control measure based on the SDQ (*r*=.20, *p*<.001). The second alternative measure consisted of a question completed by the survey interviewer assessing the extent to which the study child was focused (ranging from 1= constantly off task, to 5= constantly attends) when completing assessments at sweep 2 (i.e., standardized vocabulary and school readiness assessments). Again, there was a positive association between this alternative measure and the SDQ-based measure of self-control (*r*=.18, *p*<.001). Though correlations were modest in size we suggest this is likely due to the different raters used for each measure. For instance, a similar magnitude of association was shown between the teacher-rated and interviewer-rated measures of self-control (*r*=.18, *p*<.001) suggesting a consistent level of cross-rater correlation.

**Discriminant validity in the MCS**

Following the approach taken by Daly et al. (2015), we tested discriminant validity by examining the correlations between our self-control measure and measures of emotional functioning. We expected negative or non-significant associations between self-control and negative emotionality for two reasons: (1) Daly et al. found negative correlations between these constructs, (2) Effortful control and negative emotionality tend to be orthogonal or negatively correlated in measurement models (Rothbart, Ellis, & Posner, 2011). To facilitate comparison with the correlations reported for convergent validity, we selected indicators of emotional functioning available from teachers (three items, α=.74, e.g., ‘expresses needs and feelings in appropriate ways (reverse scored)’) and interviewers (single item: ‘extent child shows fear when approached’). The self-control measure was negatively associated with indicators of emotional difficulties as rated by teachers (*r*=-.11, *p*<.001) and not associated with emotional difficulties rated by interviewers (*r*=.01, *p*=.45).

**Testing validity in a new sample**

To test the validity of the self-control measure we conducted an online/internet study to examine its correlation with modern scales assessing children’s self/effortful control. A sample of 92 parents (95% mothers) of children aged 3 to 5 years old (*M*= 4.40, *SD*= 0.73) were recruited in collaboration with colleagues at [www.babylovesscience.com](http://www.babylovesscience.com). Parents were an average age of 36 years old (*SD*=5.64), with 48% from the UK, 39% from North America and the remainder from other English speaking (e.g., Australia) or European countries.

**Convergent validity with contemporary scales**

To test the convergent validity of our self-control measure we tested its correlation with the ‘attentional focusing’ (6 items, *α*=.73) and ‘inhibitory control’ (6 items, *α*=.70) subscales of the short-form Children’s Behaviour Questionnaire (CBQ) (Rothbart, Ahadi, Hershey, & Fisher, 2001). The CBQ is well validated and offers highly differentiated measures of children’s effortful control, in particular measures of attentional focusing (e.g., ‘when drawing or colouring in a book, shows strong concentration’) and inhibitory control (e.g., ‘can wait before entering into new activities if s/he is asked to’) (Putnam & Rothbart, 2006). Our self-control measure was positively correlated with both attentional focusing (*r*= .44, *p*<.001) and inhibitory control (*r*= .60, *p*<.001).

**Discriminant validity with contemporary scales**

Following our approach with the MCS data, we examined the discriminant validity of the self-control measure by testing its correlation with two subscales of the CBQ measuring negative affect. These were the ‘fear’ (6 items, *α*= .67, e.g., ‘is afraid of loud noises’) and ‘sadness’ (7 items, *α*=.63, e.g., ‘cries sadly when a favourite toy gets lost or broken’) subscales. The (SDQ) self-control measure showed weak negative, but non-significant, associations with both the fear (*r*= -.06, *p*=.56) and sadness (*r*= -.05, *p*=.62) scales.

Supplementary table S2.

*Indirect effects of socioeconomic factors on self-control via maternal stress*

|  | Direct effect  B [95% CI] | | Total indirect effect *via* maternal financial and emotional distress  B [95% CI] | | Total effect  B [95% CI] | |
| --- | --- | --- | --- | --- | --- | --- |
| **Socioeconomic factors** |  |  |  |  |  |  |
| Income | 0.04 [0.02, 0.07] | ^**^ | 0.02 [0.01, 0.02] | ^**^ | 0.06 [0.04,0.08] | ^**^ |
| Parental education | 0.17 [0.14, 0.21] | ^**^ | 0.00 [-0.00, 0.01] |  | 0.18 [0.14, 0.21] | ^**^ |
| Parental occupational class | 0.16 [0.11, 0.22] | ^**^ | 0.02 [0.01, 0.03] | ^**^ | 0.18 [0.13, 0.24] | ^**^ |
| Parental unemployment | -0.01 [-0.16, 0.15] |  | -0.06 [-0.09, -0.04] | ^**^ | -0.07 [-0.22, 0.09] |  |
| Single parent | 0.05 [-0.10, 0.21] |  | 0.01 [-0.01, 0.03] |  | 0.06 [-0.09, 0.22] |  |
| Mother’s age | 0.19 [0.11, 0.27] | ^**^ | -0.01 [-0.02, 0.01] |  | 0.18 [0.10, 0.27] | ^**^ |
| Family size | 0.13 [0.08, 0.17] | ^**^ | -0.01 [-0.02, -0.00] | ^**^ | 0.12 [0.07, 0.16] | ^**^ |
| Lack of home ownership | -0.23 [-0.34, -0.13] | ^**^ | -0.06 [-0.08, -0.04] | ^**^ | -0.29 [-0.40, -0.19] | ^**^ |
| Overcrowded housing | 0.01 [-0.14, 0.17] |  | -0.02 [-0.05, -0.00] | ^*^ | -0.01 [-0.17, 0.15] |  |

*Note.* ^*^ = *p*<.05, ^**^ = *p*<.01. All associations are unstandardised regression coefficients.

Model fit: Chi-square (15) = 950.478, *p*<.001. RMSEA = 0.058, sRMR = 0.014.

Supplementary table S3.

*Indirect effects of socioeconomic factors on self-control via infant temperament*

|  | Direct effect  B [95% CI] | | Total indirect effect *via* infant negative mood and withdrawal  B [95% CI] | | Total effect  B [95% CI] | |
| --- | --- | --- | --- | --- | --- | --- |
| **Socioeconomic factors** |  |  |  |  |  |  |
| Income | 0.05 [0.02, 0.07] | ^**^ | -0.00 [-0.01, 0.00] |  | 0.04, 0.02, 0.07 | ^**^ |
| Parental education | 0.19 [0.15, 0.22] | ^**^ | -0.01 [-0.02, -0.01] | ^**^ | 0.18 [0.14, 0.21] | ^**^ |
| Parental occupational class | 0.17 [0.11, 0.23] | ^**^ | -0.01 [-0.01, 0.00] |  | 0.17 [0.11, 0.22] | ^**^ |
| Parental unemployment | 0.01 [-0.15, 0.17] |  | -0.02 [-0.03, -0.00] | ^*^ | -0.00 [-0.16, 0.15] |  |
| Single parent | 0.05 [-0.11, 0.20] |  | 0.01 [-0.00, 0.02] |  | 0.06 [-0.10, 0.21] |  |
| Mother’s age | 0.19 [0.11, 0.27] | ^**^ | 0.00 [-0.01, 0.01] |  | 0.19 [0.11, 0.27] | ^**^ |
| Family size | 0.12 [0.08, 0.17] | ^**^ | 0.00 [-0.00, 0.01] |  | 0.13 [0.08, 0.17] | ^**^ |
| Lack of home ownership | -0.23 [-0.34, -0.13] | ^**^ | -0.00 [-0.01, 0.01] |  | -0.24 [-0.34, -0.13] | ^**^ |
| Overcrowded housing | 0.03 [-0.12, 0.19] |  | -0.02 [-0.04, -0.01] | ^**^ | 0.01 [-0.15, 0.17] |  |
| **Maternal subjective stress** |  |  |  |  |  |  |
| Maternal financial stress | -0.05 [-0.09, -0.01] | ^*^ | -0.01 [-0.02, -0.01] | ^**^ | -0.06 [-0.10, -0.02] | ^**^ |
| Maternal emotional distress | -.16 [-0.18, -0.14] | ^**^ | -0.03 [-0.03, -0.02] | ^**^ | -0.19 [-0.21, -0.16] | ^**^ |

*Note.* ^*^ = *p*<.05, ^**^ = *p*<.01. All associations are unstandardised regression coefficients.

Model fit: Chi-square (15) = 1749.073, *p*<.001. RMSEA = 0.079, sRMR = 0.019.

**Section 3: Results of interaction tests.**

Supplementary table S4.

*Model 4 containing interaction terms.*

|  | B | [95% CI] |  | *β* |  |
| --- | --- | --- | --- | --- | --- |
| **Socioeconomic factors** |  |  |  |  |  |
| Income | .05 | [.02, .08] |  | .04 | ^**^ |
| Parental education | .18 | [.14, .23] |  | .11 | ^***^ |
| Parental occup. class | .17 | [.10, .24] |  | .07 | ^***^ |
| Parental unemployment | .02 | [-.15, .20] |  | .00 |  |
| Single parent | .03 | [-.15, .21] |  | .01 |  |
| Mother’s age | .19 | [.09, .29] |  | .05 | ^***^ |
| Family size | .13 | [.07, .18] |  | .05 | ^***^ |
| Non home owner | -.24 | [-.36, -.12] |  | -.05 | ^***^ |
| Overcrowded housing | .05 | [-.13, .23] |  | .01 |  |
| **Maternal stress** |  |  |  |  |  |
| Financial stress | -.05 | [-.10, 00] |  | -.02 |  |
| Emotional distress | -.16 | [-.19, -.13] |  | -.12 | ^***^ |
| **Child temperament** |  |  |  |  |  |
| Negative mood | -.04 | [-.05, -.02] |  | -.08 | ^***^ |
| Withdrawal | .01 | [-.01, .03] |  | .00 |  |
| **Covariates** |  |  |  |  |  |
| Female gender | .61 | [.52, .70] |  | .13 | ^***^ |
| Birthweight | .19 | [.11, .27] |  | .05 | ^***^ |
| Ethnicity (ref: White) |  |  |  |  |  |
| Mixed | .16 | [-.10, .42] |  | .01 |  |
| Indian | -.15 | [-.47, .18] |  | -.01 |  |
| Pakistani/Bangladeshi | -.28 | [-.50, -.05] |  | -.02 | ^*^ |
| Black | .40 | [.14, .66] |  | .03 | ^**^ |
| Other | .03 | [-.45, .50] |  | .00 |  |
| **Interaction terms** |  |  |  |  |  |
| Income*Negative mood | -.01 | [-.01, .00] |  | -.03 |  |
| Education*Negative mood | .00 | [-.01, .01] |  | .00 |  |
| Occupation*Negative mood | .00 | [-.02, .01] |  | .00 |  |
| Unemployment*Negative mood | .03 | [-.01, .06] |  | .02 |  |
| Single parent*Negative mood | -.04 | [-.07, .00] |  | -.03 |  |
| Mother’s age*Negative mood | .00 | [-.02, .02] |  | .00 |  |
| Family size*Negative mood | .00 | [-.01, .01] |  | .00 |  |
| non-home owner*Negative mood | -.01 | [-.04, .02] |  | -.01 |  |
| Overcrowded*Negative mood | .01 | [-.03, .05] |  | .01 |  |
| Financial stress*Negative mood | .00 | [-.01, .01] |  | .00 |  |
| Emotional stress*Negative mood | .00 | [-.01, .01] |  | .00 |  |
| Income*Withdrawal | .00 | [-.01, .01] |  | .00 |  |
| Education*Withdrawal | -.01 | [-.02, .01] |  | -.01 |  |
| Occupation*Withdrawal | .01 | [-.01, .03] |  | .01 |  |
| Unemployment*Withdrawal | .00 | [-.05, .05] |  | .00 |  |
| Single parent*Withdrawal | .03 | [-.02, .08] |  | .02 |  |
| Mother’s age*Withdrawal | -.01 | [-.04, .02] |  | -.01 |  |
| Family size*Withdrawal | .00 | [-.02, .01] |  | .00 |  |
| Non-home owner*Withdrawal | -.02 | [-.06, .01] |  | -.03 |  |
| Overcrowded*Withdrawal | -.03 | [-.07, .02] |  | -.02 |  |
| Financial stress*Withdrawal | .01 | [-.01, .02] |  | .01 |  |
| Emotional stress*Withdrawal | .00 | [-.01, .00] |  | -.01 |  |
| *Model R^2^* |  | 12% |  |  |  |

*Notes.* ^*^ = *p*<.05, ^**^ = *p*<.01, ^***^ = *p*<.001.

**Section 4: Results of sensitivity analyses.**

**Complete cases analysis**

Supplementary Table S5. *Results of main analyses conducted on complete cases (listwise deletion)*

|  |  | Model 1 |  |  |  | Model 2 |  |  |  | Model 3 |  |  |  | Model 4 |  |  |
| --- | --- | --- | --- | --- | --- | --- | --- | --- | --- | --- | --- | --- | --- | --- | --- | --- |
|  | B | [95% CI] | *β* |  | B | [95% CI] | *β* |  | B | [95% CI] | *β* |  | B | [95% CI] | *β* |  |
| **Socioeconomic factors** |  |  |  |  |  |  |  |  |  |  |  |  |  |  |  |  |
| Income | .06 | [.03, .09] | .05 | ^***^ | .04 | [.01, .07] | .04 | ^**^ | .05 | [.02, .08] | .04 | ^**^ | .04 | [.01, .07] | .04 | ^**^ |
| Parental education | .16 | [.12, .21] | .09 | ^***^ | .16 | [.12, .21] | .09 | ^***^ | .19 | [.14, .23] | .10 | ^***^ | .18 | [.13, .23] | .10 | ^***^ |
| Parental occup. class | .19 | [.12, .26] | .07 | ^***^ | .17 | [.10, .24] | .06 | ^***^ | .17 | [.10, .24] | .07 | ^***^ | .18 | [.11, .25] | .07 | ^***^ |
| Parental unemployment | -.04 | [-.22, .15] | -.01 |  | .01 | [-.17, .20] | .00 |  | .01 | [-.16, .18] | .00 |  | .05 | [-.14, .24] | .01 |  |
| Single parent | .04 | [-.15, .23] | .01 |  | .05 | [-.14, .24] | .01 |  | .05 | [-.13, .22] | .01 |  | .02 | [-.18, .21] | .00 |  |
| Mother’s age | .18 | [.07, .29] | .04 | ^**^ | .18 | [.08, .29] | .04 | ^**^ | .19 | [.09, .29] | .05 | ^***^ | .18 | [.07, .29] | .04 | ^**^ |
| Family size | .13 | [.08, .19] | .05 | ^***^ | .14 | [.09, .20] | .06 | ^***^ | .12 | [.07, .18] | .06 | ^***^ | .14 | [.08, .20] | .06 | ^***^ |
| Non home owner | -.29 | [-.42, -.16] | -.06 | ^***^ | -.24 | [-.37, -.11] | -.05 | ^***^ | -.23 | [-.36, -.11] | -.05 | ^***^ | -.24 | [-.37, -.11] | -.05 | ^***^ |
| Overcrowded housing | -.03 | [-.22, .16] | .00 |  | .00 | [-.19, .19] | .00 |  | .03 | [-.14, .21] | .00 |  | .03 | [-.17, .23] | .00 |  |
| **Maternal stress** |  |  |  |  |  |  |  |  |  |  |  |  |  |  |  |  |
| Financial stress |  |  |  |  | -.05 | [-.10, .00] | -.02 |  | -.05 | [-.10, 00] | -.02 |  | -.05 | [-.10, .01] | -.02 |  |
| Emotional distress |  |  |  |  | -.19 | [-.22, -.16] | -.14 | ^***^ | -.16 | [-.19, -.13] | -.12 | ^***^ | -.16 | [-.19, -.13] | -.12 | ^***^ |
| **Child temperament** |  |  |  |  |  |  |  |  |  |  |  |  |  |  |  |  |
| Negative mood |  |  |  |  |  |  |  |  | -.04 | [-.05, -.03] | -.09 | ^***^ | -.04 | [-.05, -.02] | -.08 | ^***^ |
| Withdrawal |  |  |  |  |  |  |  |  | .00 | [-.01, .01] | -.01 |  | .01 | [-.01, .03] | .01 |  |
| **Covariates** |  |  |  |  |  |  |  |  |  |  |  |  |  |  |  |  |
| Female gender | .63 | [.53, .72] | .13 | ^***^ | .61 | [.52, .70] | .13 | ^***^ | .61 | [.52, .69] | .13 | ^***^ | .60 | [.50, .69] | .13 | ^***^ |
| Birthweight | .21 | [.12, .29] | .05 | ^***^ | .19 | [.11, .27] | .05 | ^***^ | .19 | [.11, .27] | .05 | ^***^ | .20 | [.11, .28] | .05 | ^***^ |
| Ethnicity (ref: White) |  |  |  |  |  |  |  |  |  |  |  |  |  |  |  |  |
| Mixed | .08 | [-.20, .36] | .01 |  | .11 | [-.16, .39] | .01 |  | .16 | [-.10, .42] | .01 |  | .15 | [-.13, .43] | .01 |  |
| Indian | -.24 | [-.58, .11] | -.01 |  | -.13 | [-.49, .23] | -.01 |  | -.15 | [-.48, .18] | -.01 |  | -.12 | [-.48, .25] | -.01 |  |
| Pakistani/Bangladeshi | -.34 | [-.59, -.09] | -.02 | ^**^ | -.32 | [-.58, -.06] | -.02 | ^*^ | -.27 | [-.50, -.04] | -.02 | ^*^ | -.32 | [-.59, -.06] | -.02 | ^*^ |
| Black | .36 | [.06, .65] | .02 | ^*^ | .38 | [.09, .67] | .02 | ^*^ | .40 | [.14, .66] | .03 | ^**^ | .46 | [.16, .76] | .03 | ^**^ |
| Other | .02 | [-.57, .61] | .00 |  | -.10 | [-.70, .50] | .00 |  | .03 | [-.44, .50] | .00 |  | -.07 | [-.68, .55] | .00 |  |
| **Interaction terms** |  |  |  |  |  |  |  |  |  |  |  |  |  |  |  |  |
| Income*Negative mood |  |  |  |  |  |  |  |  |  |  |  |  | -.01 | [-.01, .00] | -.03 |  |
| Education*Negative mood |  |  |  |  |  |  |  |  |  |  |  |  | .00 | [-.01, .01] | -.01 |  |
| Occupation*Negative mood |  |  |  |  |  |  |  |  |  |  |  |  | .00 | [-.02, .01] | -.01 |  |
| Unemployment*Negative mood |  |  |  |  |  |  |  |  |  |  |  |  | .04 | [-.00, .08] | .03 |  |
| Single parent*Negative mood |  |  |  |  |  |  |  |  |  |  |  |  | -.04 | [-.08, .00] | -.03 |  |
| Mother’s age*Negative mood |  |  |  |  |  |  |  |  |  |  |  |  | .01 | [-.02, .03] | .01 |  |
| Family size*Negative mood |  |  |  |  |  |  |  |  |  |  |  |  | .00 | [-.01, .01] | .00 |  |
| non-home owner*Negative mood |  |  |  |  |  |  |  |  |  |  |  |  | -.01 | [-.04, .02] | -.01 |  |
| Overcrowded*Negative mood |  |  |  |  |  |  |  |  |  |  |  |  | .01 | [-.03, .05] | .01 |  |
| Financial stress*Negative mood |  |  |  |  |  |  |  |  |  |  |  |  | .00 | [-.01, .01] | .00 |  |
| Emotional stress*Negative mood |  |  |  |  |  |  |  |  |  |  |  |  | .00 | [-.01, .01] | .00 |  |
| Income*Withdrawal |  |  |  |  |  |  |  |  |  |  |  |  | .00 | [-.01, .01] | .00 |  |
| Education*Withdrawal |  |  |  |  |  |  |  |  |  |  |  |  | .00 | [-.01, .01] | .00 |  |
| Occupation*Withdrawal |  |  |  |  |  |  |  |  |  |  |  |  | .01 | [-.01, .03] | .01 |  |
| Unemployment*Withdrawal |  |  |  |  |  |  |  |  |  |  |  |  | -.02 | [-.07, .03] | -.01 |  |
| Single parent*Withdrawal |  |  |  |  |  |  |  |  |  |  |  |  | .04 | [-.02, .09] | .02 |  |
| Mother’s age*Withdrawal |  |  |  |  |  |  |  |  |  |  |  |  | -.02 | [-.05, .02] | -.01 |  |
| Family size*Withdrawal |  |  |  |  |  |  |  |  |  |  |  |  | .00 | [-.02, .01] | -.00 |  |
| Non-home owner*Withdrawal |  |  |  |  |  |  |  |  |  |  |  |  | -.03 | [-.06, .01] | -.02 |  |
| Overcrowded*Withdrawal |  |  |  |  |  |  |  |  |  |  |  |  | -.02 | [-.07, .03] | -.01 |  |
| Financial stress*Withdrawal |  |  |  |  |  |  |  |  |  |  |  |  | .01 | [-.01, .02] | .01 |  |
| Emotional stress*Withdrawal |  |  |  |  |  |  |  |  |  |  |  |  | .00 | [-.01, .00] | -.01 |  |
| *Model R^2^* |  | 8% |  |  |  | 10% |  |  |  | 11% |  |  |  | 11% |  |  |
| N |  | 12917 |  |  |  | 12710 |  |  |  | 12549 |  |  |  | 12549 |  |  |

*Note.* ^*^ = *p*<.05, ^**^ = *p*<.01, ^***^ = *p*<.001.

**Analyses after dropping hyperactivity symptom items from self-control measure**

Supplementary Table S6. *Results of main analyses conducted with abbreviated self-control measure.*

|  |  | Model 1 |  |  |  | Model 2 |  |  |  | Model 3 |  |  |  | Model 4 |  |  |
| --- | --- | --- | --- | --- | --- | --- | --- | --- | --- | --- | --- | --- | --- | --- | --- | --- |
|  | B | [95% CI] | *β* |  | B | [95% CI] | *β* |  | B | [95% CI] | *β* |  | B | [95% CI] | *β* |  |
| **Socioeconomic factors** |  |  |  |  |  |  |  |  |  |  |  |  |  |  |  |  |
| Income | .03 | [.02, .05] | .05 | ^***^ | .02 | [.01, .04] | .04 | ^*^ | .03 | [.01, .04] | .04 | ^**^ | .03 | [.01, .04] | .04 | ^**^ |
| Parental education | .10 | [.07, .13] | .10 | ^***^ | .10 | [.07, .13] | .10 | ^***^ | .10 | [.08, .13] | .10 | ^***^ | .10 | [.08, .13] | .10 | ^***^ |
| Parental occup. class | .06 | [.01, .10] | .04 | ^*^ | .05 | [.00, .09] | .03 | ^*^ | .05 | [.01, .09] | .03 | ^*^ | .05 | [.01, .09] | .03 | ^*^ |
| Parental unemployment | .04 | [-.06, .15] | .01 |  | .07 | [-.03, .18] | .02 |  | .08 | [-.02, .19] | .02 |  | .08 | [-.02, .19] | .02 |  |
| Single parent | .06 | [-.05, .17] | .02 |  | .06 | [-.05, .16] | .01 |  | .05 | [-.06, .16] | .01 |  | .04 | [-.07, .15] | .01 |  |
| Mother’s age | .13 | [.06, .19] | .05 | ^***^ | .13 | [.07, .19] | .05 | ^***^ | .13 | [.07, .19] | .05 | ^***^ | .13 | [.07, .19] | .05 | ^***^ |
| Family size | .03 | [-.01, .06] | .02 |  | .03 | [-.00, .06] | .02 |  | .03 | [-.01, .06] | .02 |  | .03 | [-.01, .06] | .02 |  |
| Non home owner | -.07 | [-.15, .00] | -.03 |  | -.04 | [-.12, .03] | -.02 |  | -.04 | [-.12, .03] | -.02 |  | -.05 | [-.13, .03] | -.02 |  |
| Overcrowded housing | .01 | [-.10, .12] | .00 |  | .02 | [-.09, .13] | .00 |  | .03 | [-.08, .14] | .01 |  | .04 | [-.08, .15] | .01 |  |
| **Maternal stress** |  |  |  |  |  |  |  |  |  |  |  |  |  |  |  |  |
| Financial stress |  |  |  |  | -.05 | [-.08, -.02] | -.03 | ^**^ | -.04 | [-.08, -.01] | -.03 | ^**^ | -.04 | [-.08, -.01] | -.03 | ^**^ |
| Emotional distress |  |  |  |  | -.07 | [-.09, -.06] | -.09 | ^***^ | -.06 | [-.08, -.04] | -.07 | ^***^ | -.06 | [-.08, -.04] | -.07 | ^***^ |
| **Child temperament** |  |  |  |  |  |  |  |  |  |  |  |  |  |  |  |  |
| Negative mood |  |  |  |  |  |  |  |  | -.03 | [-.03, -.02] | -.09 | ^***^ | -.02 | [-.03, -.01] | -.08 | ^***^ |
| Withdrawal |  |  |  |  |  |  |  |  | .00 | [-.00, .01] | .01 |  | .01 | [-.00, .02] | .02 |  |
| **Covariates** |  |  |  |  |  |  |  |  |  |  |  |  |  |  |  |  |
| Female gender | .39 | [.34, .45] | .13 | ^***^ | .39 | [.33, .44] | .13 | ^***^ | .38 | [.33, .43] | .14 | ^***^ | .38 | [.33, .44] | .14 | ^***^ |
| Birthweight | .11 | [.06, .16] | .05 | ^***^ | .11 | [.06, .15] | .5 | ^***^ | .11 | [.06, .16] | .05 | ^***^ | .11 | [.06, .16] | .05 | ^***^ |
| Ethnicity (ref: White) |  |  |  |  |  |  |  |  |  |  |  |  |  |  |  |  |
| Mixed | -.01 | [-.17, .15] | .00 |  | .01 | [-.14, .17] | .00 |  | .04 | [-.12, .19] | .00 |  | .04 | [-.12, .20] | .01 |  |
| Indian | .08 | [-.13, .28] | .01 |  | .12 | [-.10, .33] | .01 |  | .11 | [-.10, .33] | .01 |  | .11 | [-.10, .33] | .01 |  |
| Pakistani/Bangladeshi | .00 | [-.15, .15] | .00 |  | .03 | [-.12, .18] | .00 |  | .05 | [-.10, .20] | .01 |  | .04 | [-.11, .19] | .01 |  |
| Black | .17 | [.00, .34] | .02 | ^*^ | .20 | [.03, .37] | .02 | ^*^ | .24 | [.07, .40] | .03 | ^**^ | .25 | [.08, .41] | .03 | ^**^ |
| Other | .05 | [-.25, .35] | .00 |  | .04 | [-.26, .34] | .00 |  | .09 | [-.21, .38] | .01 |  | .09 | [-.20, .39] | .01 |  |
| **Interaction terms** |  |  |  |  |  |  |  |  |  |  |  |  |  |  |  |  |
| Income*Negative mood |  |  |  |  |  |  |  |  |  |  |  |  | -.00 | [-.01, .00] | -.02 |  |
| Education*Negative mood |  |  |  |  |  |  |  |  |  |  |  |  | .00 | [-.01, .01] | .00 |  |
| Occupation*Negative mood |  |  |  |  |  |  |  |  |  |  |  |  | .00 | [-.02, .01] | .00 |  |
| Unemployment*Negative mood |  |  |  |  |  |  |  |  |  |  |  |  | .02 | [-.00, .04] | .03 |  |
| Single parent*Negative mood |  |  |  |  |  |  |  |  |  |  |  |  | -.02 | [-.04, .01] | -.02 |  |
| Mother’s age*Negative mood |  |  |  |  |  |  |  |  |  |  |  |  | -.01 | [-.02, .01] | -.01 |  |
| Family size*Negative mood |  |  |  |  |  |  |  |  |  |  |  |  | .00 | [-.00, .01] | .01 |  |
| non-home owner*Negative mood |  |  |  |  |  |  |  |  |  |  |  |  | -.01 | [-.03, .01] | -.03 |  |
| Overcrowded*Negative mood |  |  |  |  |  |  |  |  |  |  |  |  | .01 | [-.02, .03] | .01 |  |
| Financial stress*Negative mood |  |  |  |  |  |  |  |  |  |  |  |  | .00 | [-.01, .01] | -.00 |  |
| Emotional stress*Negative mood |  |  |  |  |  |  |  |  |  |  |  |  | .00 | [-.00, .01] | .02 |  |
| Income*Withdrawal |  |  |  |  |  |  |  |  |  |  |  |  | -.00 | [-.01, .00] | -.01 |  |
| Education*Withdrawal |  |  |  |  |  |  |  |  |  |  |  |  | -.00 | [-.01, .00] | -.01 |  |
| Occupation*Withdrawal |  |  |  |  |  |  |  |  |  |  |  |  | -.00 | [-.02, .01] | -.01 |  |
| Unemployment*Withdrawal |  |  |  |  |  |  |  |  |  |  |  |  | .00 | [-.03, .03] | .00 |  |
| Single parent*Withdrawal |  |  |  |  |  |  |  |  |  |  |  |  | .02 | [-.01, .04] | .02 |  |
| Mother’s age*Withdrawal |  |  |  |  |  |  |  |  |  |  |  |  | .00 | [-.02, .02] | .00 |  |
| Family size*Withdrawal |  |  |  |  |  |  |  |  |  |  |  |  | -.00 | [-.01, .01] | -.01 |  |
| Non-home owner*Withdrawal |  |  |  |  |  |  |  |  |  |  |  |  | -.01 | [-.04, .01] | -.02 |  |
| Overcrowded*Withdrawal |  |  |  |  |  |  |  |  |  |  |  |  | -.01 | [-.03, .02] | -.01 |  |
| Financial stress*Withdrawal |  |  |  |  |  |  |  |  |  |  |  |  | .00 | [-.01, .01] | .01 |  |
| Emotional stress*Withdrawal |  |  |  |  |  |  |  |  |  |  |  |  | -.00 | [-.01, .00] | -.01 |  |
| *Model R^2^* |  | 6% |  |  |  | 7% |  |  |  | 8% |  |  |  | 8% |  |  |

*Note.* ^*^ = *p*<.05, ^**^ = *p*<.01, ^***^ = *p*<.001.

**References**

Daly, M., Delaney, L., Egan, M., & Baumeister, R. F. (2015). Childhood self-control and unemployment throughout the life span: Evidence from two british cohort studies. *Psychological Science, 26*, 709-723.

Putnam, S. P., & Rothbart, M. K. (2006). Development of short and very short forms of the children's behavior questionnaire. *Journal of Personality Assessment, 87*, 102-112.

Rothbart, M. K., Ahadi, S. A., Hershey, K. L., & Fisher, P. (2001). Investigations of temperament at three to seven years: The children's behavior questionnaire. *Child Development, 72*, 1394-1408.

Rothbart, M. K., Ellis, L. K., & Posner, M. I. (2011). Temperament and self-regulation. In K. D. Vohs & R. F. Baumeister (Eds.), *Handbook of self-regulation: Research, theory, and applications* (2nd edition ed., pp. 441-460). New York: Guilford Press.
